# Corona discharge plasma for green de-inking of inkjet printer ink

**DOI:** 10.1038/s41598-024-63683-8

**Published:** 2024-06-06

**Authors:** Ika Priyanti, Doonyapong Wongsawaeng, Kanokwan Ngaosuwan, Worapon Kiatkittipong, Peter Hosemann, Suttichai Assabumrungrat

**Affiliations:** 1 Research Unit on Plasma Technology for High-Performance Materials Development, Department of Nuclear Engineering, Faculty of Engineering, Chulalongkorn University, 254 Phayathai Road, Pathumwan, Bangkok, 10330 Thailand; 2 Division of Chemical Engineering, Faculty of Engineering, Rajamangala University of Technology Krungthep, Bangkok, 10120 Thailand; 3 Department of Chemical Engineering, Faculty of Engineering and Industrial Technology, Silpakorn University, Nakhon Pathom, 73000 Thailand; 4 Department of Nuclear Engineering, Faculty of Engineering, University of California at Berkeley, Berkeley, 94720 USA; 5 Center of Excellence in Catalysis and Catalytic Reaction Engineering, Department of Chemical Engineering, Faculty of Engineering, Chulalongkorn University, Bangkok, 10330 Thailand; 6 Bio-Circular-Green-Economy Technology and Engineering Center (BCGeTEC), Faculty of Engineering, Chulalongkorn University, Bangkok, 10330 Thailand

**Keywords:** Corona discharge plasma, Green de-inking, Paper recycling, Chemical engineering, Sustainability

## Abstract

This work features a new corona discharge plasma technology for de-inking yellow, blue, and red colors on various papers. This work was developed to minimize the chemical and environmental impacts of de-inking processes. A nonchemical contribution, operating at room temperature and atmospheric pressure, reduces the environmental impact of the process. The deinkability factor (DEM_Lab_) values for all papers are determined with the optimal assessment results provided by a 36-mm variation gap at 2-min (blue) and 10-min (yellow and red) plasma exposure times, followed by applied voltages of 20 kV (yellow), 16 kV (blue), and 20 kV (red). The corona discharge plasma led to 48.58% (yellow printed paper), 64.11% (blue printed paper), and 41.11% (red printed paper) deinkability without altering the physical properties of the paper itself. The change in the tensile strength for the plasma-exposed paper was relatively little, less than 10%, compared to that of common recycling. The tensile strength of the untreated white paper was 5065 ± 487.44 N/mm^2^, and that of the plasma-treated printed paper was 4593 ± 248.47 N/mm^2^. It appears that there is little impact on the physicochemical properties of paper induced by the corona plasma treatment during the de-inking process.

## Introduction

Recycling reduces energy use and saves landfills, making the future greener and more sustainable^1^. Through efficient recycling systems, the recyclable material lifecycle can be extended, and the adverse environmental impact can be minimized. Various environmental initiatives and sustainable practices aim to preserve virgin material sources and reduce the massive volume of waste entering landfills, particularly within the realm of paper recycling. Paper recycling technology today can be categorized into two types: conventional and waterless technology^2^. The massive water consumption of the conventional method produces additional operational costs for wastewater treatment. In addition, chemical ink, e.g., azo dyes, can cause imbalanced biodiversity and ameliorate human health. Azo dyes are aromatic compounds usually found in printed paper ink that pose a carcinogenic risk to the human body through ingestion or skin contact^3^. This leads to waterless technology being more environmentally friendly since water resources are conserved. However, the related technology requires large amounts of capital and operational investments due to the high electrical demands to support costly equipment^4^, making this a non-competitive approach.

De-inking is a pretreatment process through which color pigments are removed during every recycling step so that the paper can be further processed. Typically, this process uses surface active agents to enhance the efficiency of the flotation process. Methods for de-inking, ranging from bacterial cellulase, chemical, enzymatic, to ozone-based approaches, have been employed^5–7^. While ozone-based treatment is widely utilized for de-coloring processes due to its effectiveness in breaking down organic compounds, it presents drawbacks such as reliance on pure oxygen, and high overhead^8^. Additionally, the generated byproducts (namely bromate and nitrosamines) may require further processing or disposal, adding to operational expenses and environmental considerations due to they are carcinogenic to humans^8^. Other approaches may be more resource-intensive and may require further preconditions or incubation processes (like the bacterial process). It is crucial to develop novel approaches that effectively address these concerns to guarantee a sustainable and environmentally friendly future for the paper manufacturing and recycling industry. One proposed method for addressing this problem is plasma de-inking. Plasma de-inking utilizes an ionized gas to create a radical environment. These reactive species are used to enhance chemical reactions. Plasma de-inking offers potential advantages in terms of cost-effectiveness and environmental impact due to utilizing the most abundant gas: air. Its ability to efficiently degrade dyes without the need for chemical additives or extensive post-treatment processes could result in lower operational costs and reduced environmental footprint. Also, corona plasma systems are often more compact and require less maintenance compared to ozone systems. In contrast, certain chemicals such as NaOH, Na_2_SiO_3_, H_2_O_2_, and pentetic acid are commonly utilized in conventional chemical de-inking processes, to create alkaline environments^9^.

Reported plasma generates various reactive oxygen and nitrogen species (RONS), such as ozone (O_3_), nitric oxide (NO), nitrite (NO_2_^−^), nitrate (NO_3_^−^), hydroxyl (⋅OH), hydroperoxide (⋅HO_2_), hydrogen peroxide (H_2_O_2_), and peroxynitrite (ONOO^−^), which have attracted some researchers to use this method due to their high oxidation potentials to effectively react with pollutant molecules^10^. In nonthermal plasma (NTP), although the electron temperature increases from 10^4^ to 10^5^ K, the gas temperature remains ambient^11^. One discharge type is corona discharge plasma. This versatile technology plays a pivotal role in various aspects of life. Corona discharge plasma purifies the air by removing pollutants such as formaldehyde and benzene from burning fuel in air purifier applications^12,13^. In the energy sector, it facilitates the conversion of frying oil into biodiesel^14^. In agriculture, corona discharge plasma enhances the quality and shelf life of cherry tomatoes and improves the germination of sunflower seeds^15,16^. Moreover, this technology modifies the physicochemical properties of banana starch, increasing the gelatinization temperature and crystallinity^17^. Additionally, it is utilized for sterilization and microbial decontamination of dried squid, for viral inactivation (including SARS-CoV-2), and water purification from active pharmaceutical compounds (PhAcs)^18–20^. In environmental applications, corona discharge plasmas remove antibiotics and degrade pollutants, such as gasoline and nitrogen, and remediate perfluorooctanoic acid (PFOA) from soil^21–23^. These interconnected uses of corona plasmas create a compelling narrative of its diverse and impactful role in some lives.

According to Hafeez et al.^24^, air corona plasmas form various generations of diverse active species. Corona plasma reactors usually use free electrons with an average energy of 1–10 eV, and they lose their energy through collisions with the paper surface. The electrons in corona plasma reactors carry sufficient energy to break the bond energies of C–H (4.36 eV), C–O (3.71 eV), and O–H (4.43 eV)^25^. The resulting free radicals lead to the breaking of cellulose fibers and the opening of pores in the material. Surface hydrophilicity improvement has been previously reported using plasma treatments with various carrier gases, including argon, helium, nitrogen, oxygen, and air^26^. Because hydrophilicity improves the wetting of paper, it can play an essential role in the swelling of fibers, impacting the efficiency of the de-inking process. Considering the abovementioned mechanisms, researchers are interested in the role of corona plasma in paper de-inking.

The true strength of corona plasma de-inking occurs through a one-step process without the use of further chemicals. To our knowledge, no previous studies have evaluated the impact of corona plasma treatment on the de-inking of inkjet-printed paper. The objectives of the present study are to evaluate the ability of corona plasma for de-inking paper as a function of de-inking parameters and the reliability and accuracy of brightness methods.

## Materials and methods

### Materials

A4 paper sheets (Double-A, 80 g/m^2^, Thailand) were purchased and printed using an inkjet printer (Epson Eco Tank L3150 Wi-Fi All-in-One Ink Tank Printer, Thailand) with a Micro Piezo™ print head (cartridge-free printing). The paper sheets were printed in red, blue, and yellow (Epson Eco Tank Printer Ink 003, Thailand). Printing inks can comprise pigments/dyes (Cu, Fe, Cr, and Ba), inorganic extenders (Al, Mg, Ca, Si, and Ti), driers (Co, Ce, W, Zr, and Pb), and/or other additives (Li, Si, Zn, and Mg) that affect a certain ink feature^28^. The chemical compositions of red, blue, and yellow ink used in Epson inkjet printing have been reported previously^29^. The red color contained 2-Naphthalenol (commonly known as pigment red, C_18_H_16_N_2_O), the blue color contained copper phthalocyanine (C_32_H_15_N_8_Cu/C_32_H_16_N_8_), and the yellow color contained pigment yellow (C_12_H_8_Br_2_N_2_).

### Experimental setup and procedure

**Figure 1:**
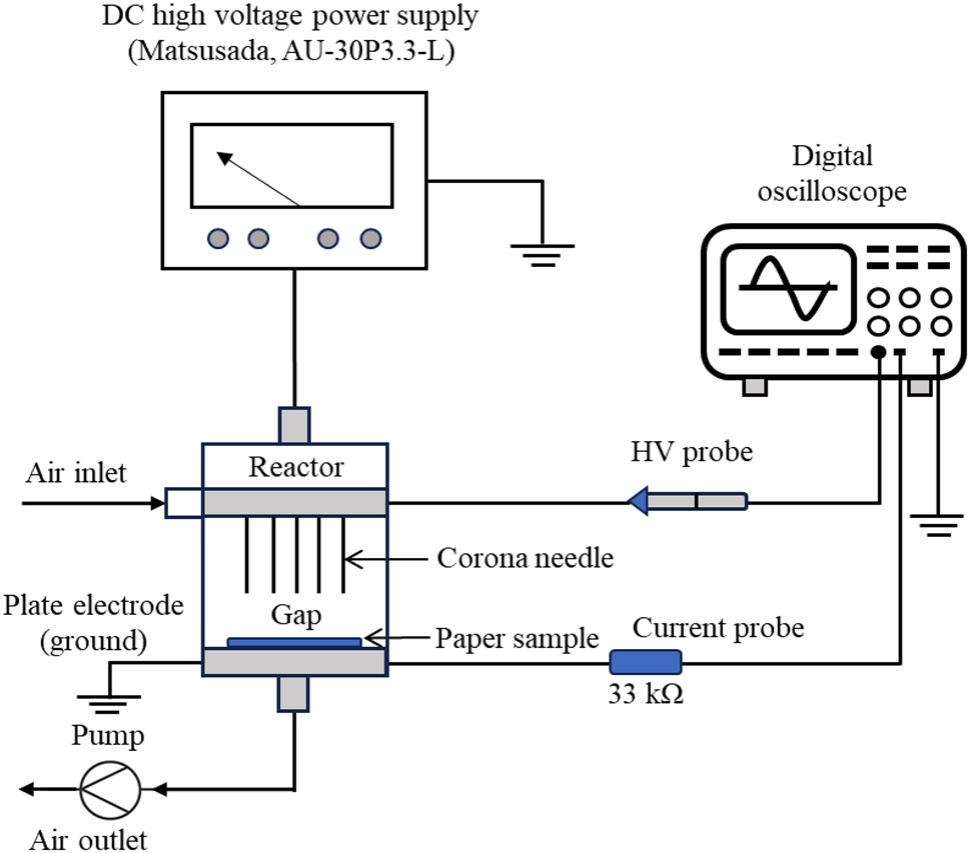
Schematic of experimental setup for corona discharge plasma system, which contains plasma reactor, DC high-voltage power supply, and electrical measurement system.

Plasma treatment was performed using an atmospheric pressure corona discharge plasma, as illustrated in Fig. 1, consisting of an adjustable high-voltage positive direct current (HVDC) power supply, a corona discharge reactor, and an electrical circuit for measurement. The HVDC power supply (MATSUSADA Precision, Model AU-30P3.3-L, Japan) could provide a high voltage range output of 0–30 kV (positive polarity, 3.3 mA maximum, 100 W maximum) and was used to drive the discharge. The gap between the edge of the needle tip and the ground plate electrode surface was measured.

The paper sample was fixed on an aluminum ground electrode. Air at atmospheric pressure was used as a working gas in all the experiments; the corona was characterized by measuring the discharge voltage and discharge current using a digital oscilloscope (Tektronix TDS 2012). The oscilloscope was equipped with an HV probe (Keithley 1600A series, USA), a voltage probe (Tektronix, P2220), and a 33 kΩ resistor for current probe measurements, as described in prior work^30^. The yellow, blue, and red printed paper dimensions were 5 cm × 5 cm for all the experiments. The effects of the specific experimental parameters on the printed paper are listed in Table 1.

**Table 1:** Variations in the experimental parameters to obtain optimal results.

Batch	Applied voltage (kV)			Treatment time (min)			Gap (mm)
1	(All paper)						
	16			10			36
	18			10			36
	20			10			36
	22			10			36
	24			10			36
2^a^	Yellow	Blue	Red				
	20	16	20	2			36
	20	16	20	4			36
	20	16	20	6			36
	20	16	20	8			36
	20	16	20	10			36
	20	16	20	15			36
	20	16	20	20			36
3^b^	(All paper)			Yellow	Blue	Red	
	6			10	2	10	6
	10			10	2	10	11
	12.5			10	2	10	16
	24			10	2	10	36

^a^Optimal applied voltage was used based on the deinkability factor results of the 1st batch.

^b^Gap variation with respect to the maximum breakdown HV value.

### De-inking protocols

The effect of plasma power on de-inking performance was studied in several iterations. The experimental matrix aimed to study the effect of the plasma gap and exposure time. The gap experiment involved a plasma exposure time of 10 min on a 36 mm gap between printer papers with various voltages of 16, 18, 20, 22, and 24 kV and direct immersion in 100 mL of water for 1 h in a closed container, after which the samples were subsequently air-dried within 24 h. The applied voltages were recorded until the plasma disappeared, which allowed us to provide the maximum breakdown voltage for the gap. The exposure time experiment aimed to study the effects of plasma exposure times of 2, 4, 6, 8, 10, 15, and 20 min for a 36 mm gap; subsequently, the samples were exposed to an applied voltage of 20 kV for the yellow and red printed paper and 16 kV for the blue printed paper from the abovementioned gap experiment. The following experiment studied gap sizes of 6, 11, 16, and 36 mm using a plasma exposure time of 10 min for yellow and red printer paper and 2 min for blue printed paper. All the experiments were performed in triplicates to ensure that statistical error could be accounted for.

### Paper colorimeter

To determine the extent of the de-inking, the difference in color between the samples was measured before and after the de-inking process using a portable colorimeter (VINCKOLOR PRO, China). A colorimeter was used to quantify the surface L*, a*, and b* values. The CIELab color space is frequently employed as a way of characterizing colors. L* corresponds to the brightness, with L* = 100 to 0 representing the continuum from white to black. The a* value corresponds to a scale ranging from green to red, with the highest positive value to the lowest negative value corresponding to the red–green gradient. The b* value corresponds to a blue‒yellow gradient, with the highest positive value corresponding to yellow and the lowest negative value corresponding to blue. Deinkability was evaluated by the deinkability factor (**DEM**_**Lab**_) on plasma-treated paper and untreated paper, which represents the complete removal of color printing ink. This method was developed by the previous laboratory of the Department of Paper Science and Technology (IfP), Darmstadt, Germany, and the Papiertechnische Stiftung (PTS), Munich, Germany. DEM_Lab_ is expressed as a percentage (Eq. 1)^31^.

1 $${DEM}_{Lab}= \left(1- \sqrt{\frac{{\left({L}_{US}^{*}- {L}_{DS}^{*}\right)}^{2}+{\left({a}_{US}^{*}- {a}_{DS}^{*}\right)}^{2}+{\left({b}_{US}^{*}- {b}_{DS}^{*}\right)}^{2}}{{\left({L}_{US}^{*}- {L}_{BS}^{*}\right)}^{2}+{\left({a}_{US}^{*}- {a}_{BS}^{*}\right)}^{2}+{\left({b}_{US}^{*}- {b}_{BS}^{*}\right)}^{2}}}\right)\times 100 \%$$

where L*_US_, a*_US_, and b*_US_ are the color coordinates of unprinted/initial paper; L*_BS_, a*_BS_, and b*_BS_ are the color coordinates of plasma-treated printed paper; and L*_DS_, a*_DS_, b*_DS_ are the color coordinates of plasma-treated printed paper that is deinked via water immersion. Initial papers were used as a reference, while a 100% value of **DEM**_**Lab**_ was used to describe the complete removal of printing ink.

To provide insight into the influences of residual ink on paper on changes in light reflectance at wavelengths ranging from 400 to 800 nm, the samples were characterized by ultraviolet (UV)‒visible (vis) spectroscopy (Genesys 180, USA)^31^. ATR-FTIR spectroscopy was utilized the Bruker ATR-FTIR spectrometer (Bruker Corporation, Massachusetts, U.S.A.) in the middle infrared mode equipped with an ATR sampling device. Spectra were acquired within a wavenumber range of 4000–500 cm^−1^ with 16 scans at 4 cm^−1^ resolution. A method to characterize printing papers was used a positive ion DART-MS which followed the previous report^32^. The spectra were analyzed using NIST MS Search v.2.3 and Mestrenova software. The paper surface was examined using scanning electron microscopy (SEM) (JEOL, Model JSM-IT300, Japan). Tensile strength tests were performed using a UTM1T universal testing machine (manufactured by Dartec, Model H10 KM). A static optical contact angle meter (KINO, Model SL150E, USA) was used to determine the optimal treatment time for the plasma exposure-time parameter. In this standard method, a liquid droplet was dispensed on a solid surface. A diffuse light source illuminated the sessile drop from one side, and the camera captured the contour of the water drop image from the other side. This characterization was performed as described in a previous investigation^33^. Smaller contact angles indicated higher wettability and hydrophilic character.

## Results and discussion

### Electrical characteristics of the corona discharge plasma

**Figure 2:**
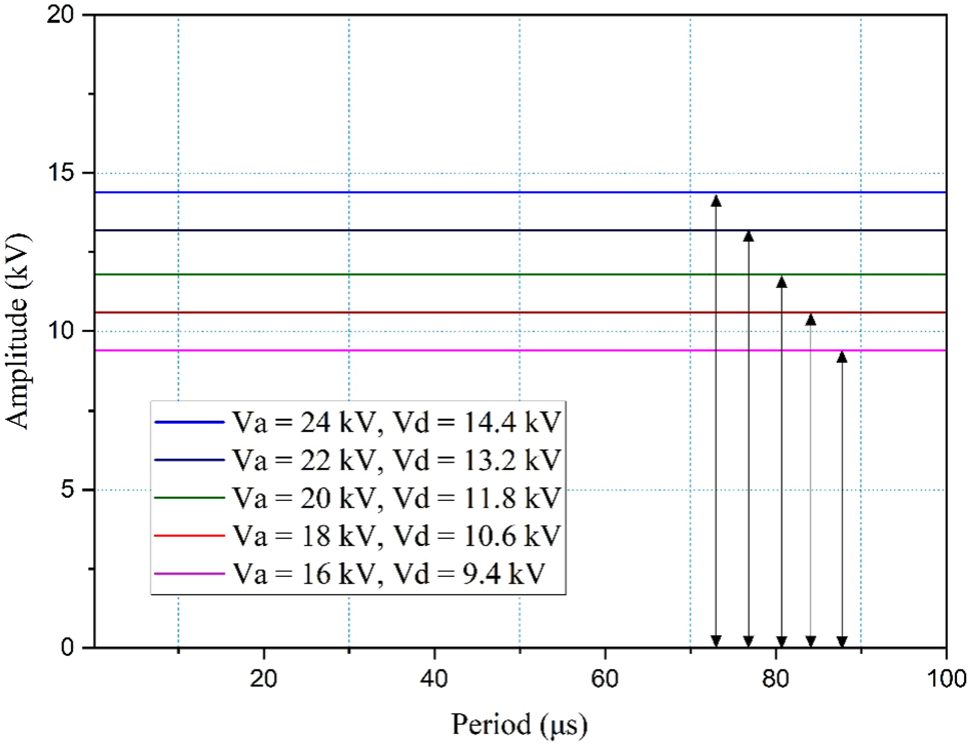
The schematic representations of voltage signals from corona discharge plasma on yellow printed paper under V_a_ values of 16, 18, 20, 22, and 24 kV and V_d_ values of 9.4, 10.6, 11.8, 13.2, and 14.4 kV (the signals of corona discharge plasma on red and blue printed papers are presented in the Tables S2 and S3).

The voltage between an applied voltage and a discharge voltage is measured to provide essential information about the electrical behavior of the corona plasma. The experiment was conducted with a gap length of 36 mm, a plasma exposure time of 10 min, and an immersion time of 1 h. Figure 2 shows the typical DC signals of the discharge voltage of the corona discharge plasma using air as the carrier gas. Pulses with applied voltage (V_a_) values of 16, 18, 20, 22, and 24 kV were observed in the DC signals of the yellow printed paper treated with plasma, with maximum discharge voltage (V_d_) values of 9.40, 10.6, 11.8, 13.2, and 14.4 kV.

The voltage drop from a V_a_ of 16 kV to the V_d_ was 9.4 kV, as shown in Fig. 2. The 6.6-kV energy loss (from 16 to 9.4 kV) likely occurred due to material erosion^34,35^. Material erosion during corona discharge was studied by Cogollo et al*.*^34^ focusing on the erosion mechanism and corona filament oxidation process. It was further found that the immediate breakage of corona electrodes with a further high-voltage drop may be due to stainless steel materials. The low boiling temperatures of the constituent alloy material were lower (< 10^4^ K) compared to the temperatures of generated corona plasma (10^4^ to 10^5^ K)^34^. Also, a higher temperature difference leads to a more rapid heat transfer through conduction, and the material undergoes structural changes to experience such erosion or cracking.

**Figure 3:**
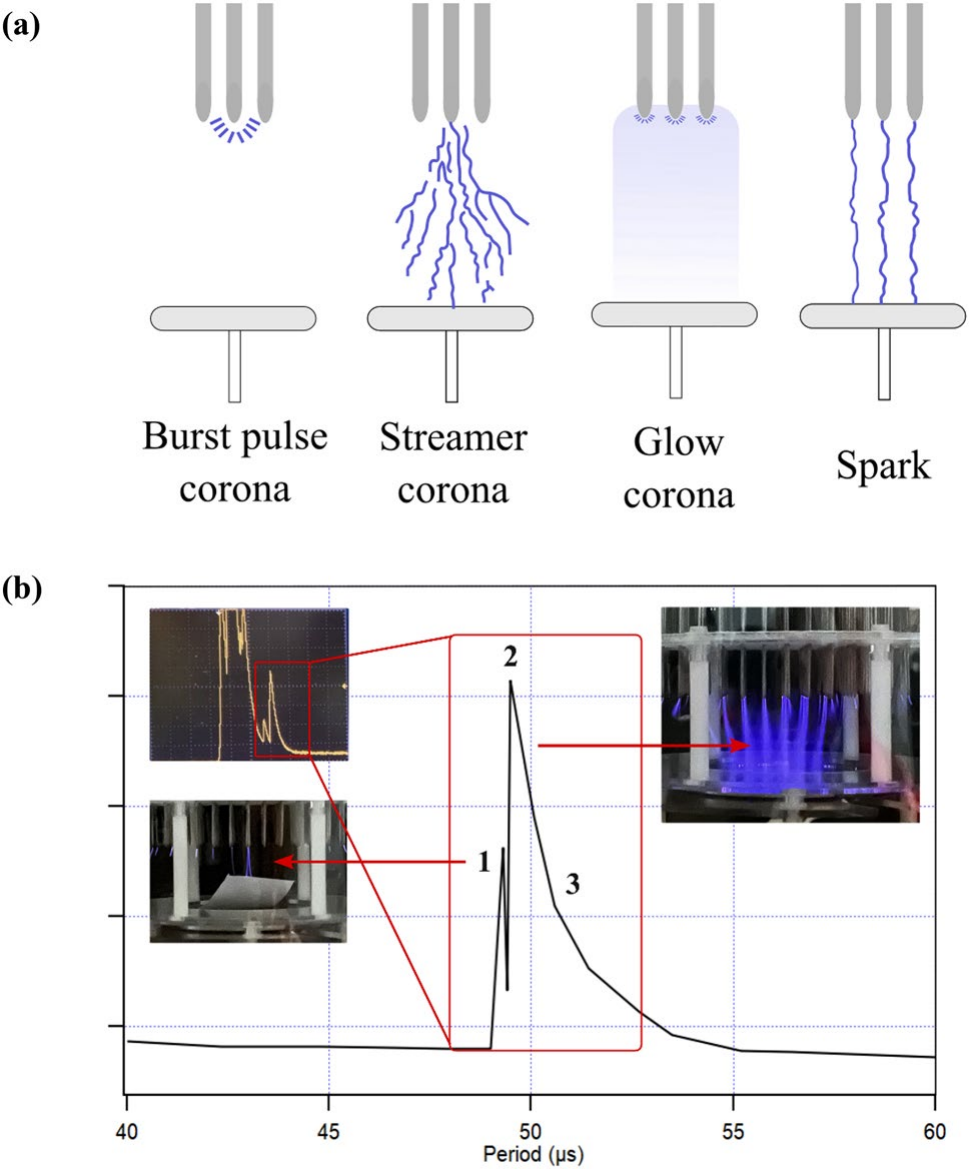
(**a**) Schematic of several types of corona discharge (modified from Chang et al.^40^), (**b**) current signal representation of corona discharge plasma on yellow printed paper under a V_a_ of 24 kV, a V_d_ of 14.4 kV (i_d_ = 1.37 A), a gap length of 36 mm, a plasma exposure time of 10 min, and an immersion time of 1 h.

Furthermore, a voltage drop was observed in the electrical equivalent circuit of the pin-to-plate corona plasma. Saber et al.^36^ also reported that the energy efficiency was lower than 50% with a V_a_ maximum of 9 kV. In addition, this work showed 58.75% energy efficiency. These findings suggest that the higher V_a_ used in this work (16 kV compared to that used by Sabel et al. at 9 kV) improved the efficiency of the process. Efficiency was calculated with Eq. (2)^37^:

2 $$\upeta \left(\%\right)= \frac{{P}_{dis}}{{P}_{d}}\times 100 \%$$

where P_dis_ is the discharge power and P_d_ is the power delivered to the reactor during discharge. P_dis_ is calculated by multiplying the V_d_ (9.4 kV) as shown in Fig. 2 and the calculated current of 0.67 A (*i* = V/R = 22.4 kV/33 kΩ = 0.67 A) from the observed voltage as shown in Fig. 3b. P_d_ is calculated by multiplying the V_a_ (16 kV) as shown in Fig. 2 and the calculated current of 0.67 A (*i* = V/R = 22.4 kV/33 kΩ = 0.67 A) from the observed voltage, as mentioned previously. The 58.75% energy efficiency was calculated by dividing the discharge power by the delivered power (9.4 kV/16 kV). Several authors, such as An et al. and Wen et al*.*^38,39^, considered affecting the average electron energy and the effective ionization rate in an atmospheric air-corona plasma. This was speculated to be the cause of the power loss since a reduced voltage is correlated with reduced power. Atmospheric air is commonly known as an electronegative gas, wherein both ionization and electron attachment processes occur simultaneously.

Figure 3a shows different kinds of corona discharge that depend on the strength of the field and the shape of the electrodes. Based on the findings of Chang et al.^40^, as the applied voltage increases, the needle-plate electrode starts to discharge with the burst pulse corona and then moves on to the streamer corona, glow corona, and spark discharge. During the plasma treatment, three distinct stages are observed for which the current is evaluated (Fig. 3b): (1) the main streamer spreads between the needle tip and the ground electrode through ionization, (2) the maximum current is reached when the ground electrode absorbs the accumulated charge and the discharge turns to a glow corona, and (3) the electrons in the plasma area leave by recombining with the surrounding area or when the stored electrons start moving toward the ground cable in the closed circuit, which is called the relaxation phase or discharging moment. These findings contribute to a comprehensive understanding of the dynamic stages during corona discharge.

**Figure 4:**
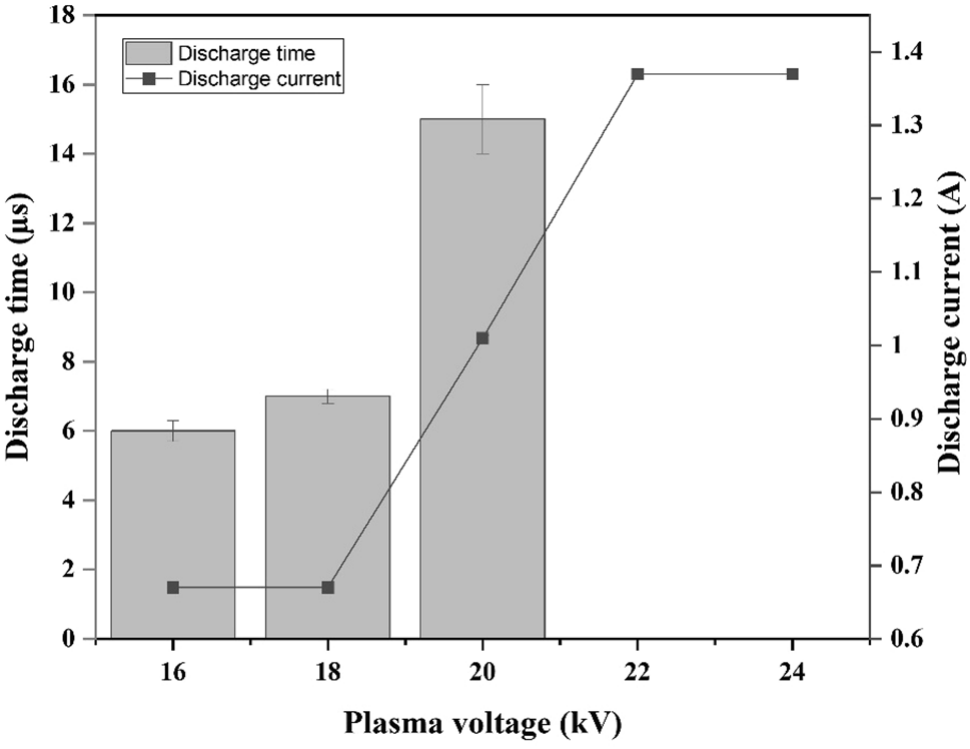
Discharge current and discharge time over V_a_ variations for all printed paper.

Figure 4 summarizes the trend of electrical pulses of all printed paper under V_a_ values of 16, 18, 20, 22, and 24 kV, with the maximum discharge current amplitude (i_d_) values of the yellow-printed paper being recorded as 0.67, 0.67, 1.01, 1.37, and 1.37 A, respectively. The discharge current increases significantly when the voltage is raised more than 18 kV.

The discharge time of all printed paper was observed and recorded as 6, 7 and 15 µs for V_a_ of 16, 18 and 20 kV. The values markedly unidentified at V_a_ of 22 kV and 24 kV. This observation was happened due to the limitations of instrument which depicted the clipped current signal at V_a_ of 22 kV and 24 kV (see Table S4).

### Performance of the applied voltage to the L*-a*-b* coordinates and DEM_Lab_.

**Figure 5:**
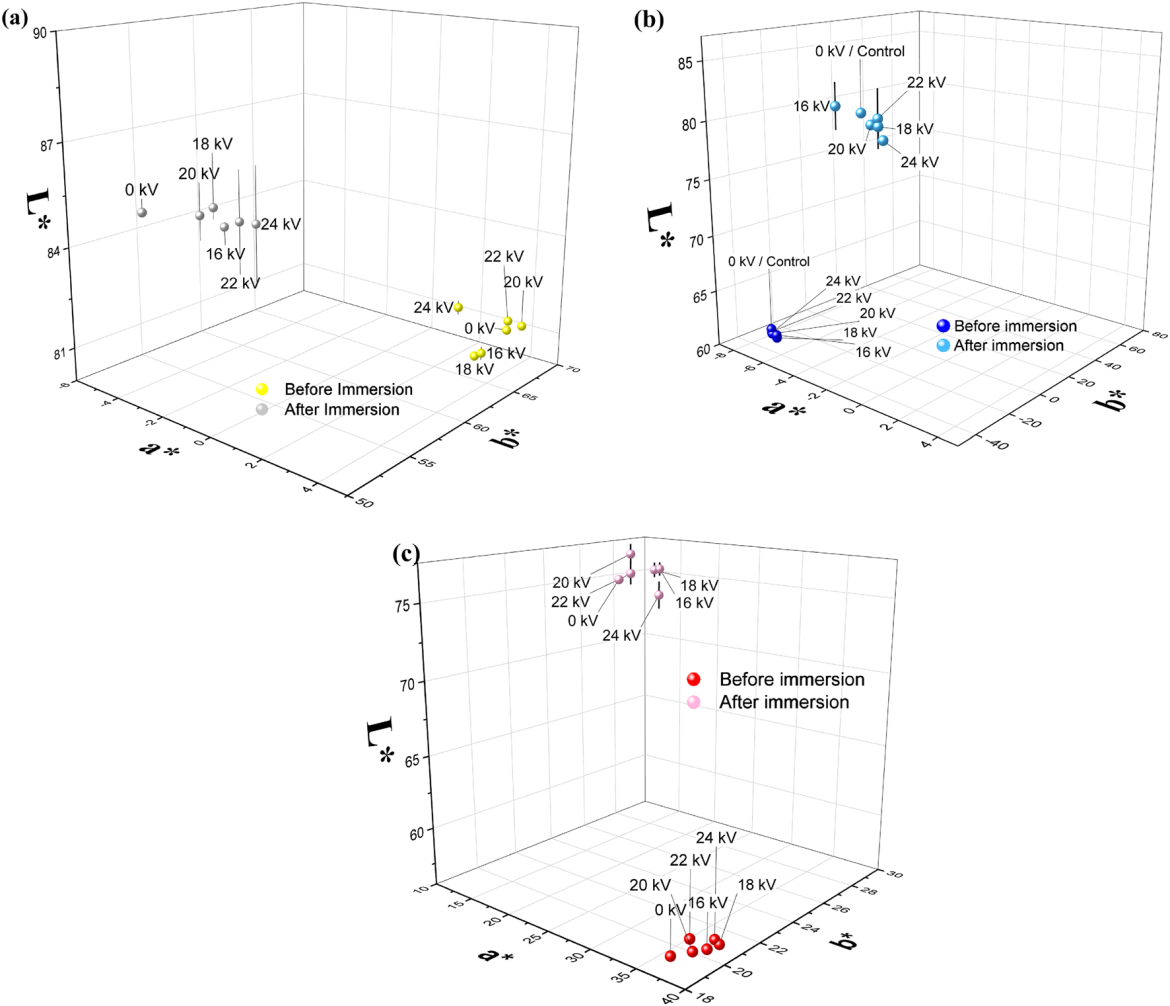
L*-, a*-, and b* color coordinates before and after 1 h of immersion in water under various applied voltages for (**a**) yellow, (**b**) blue, and (**c**) red printed paper.

As shown in Eq. (1) as method section "Paper colorimeter", the color space factors L*, a*, and b* allow us to calculate the factor **DEM**_**Lab**_, which is a measure of de-inking. A colorimeter giving the L*-, a*-, and b* color values was used to quantify the observed color alterations in the plasma-treated regions. Figure 5a–c show the L*-, a*-, and b* color coordinates for the treated paper before and after the water immersion stage. The immersion stage exhibits a slight increase in the L* value, as shown in Fig. 5a–c, suggesting that the sample is lighter in color. The V_a_ values are 0, 16, 18, 20, 22, and 24 kV. The plasma exposure time was set at 10 min. These variations suggest that the implementation of plasma treatment increases paper brightness and reduces paper color values, thus improving the ink removal efficiency relative to that without plasma treatment under those conditions. This behavior shows that the voltage variation impacts the colorization pathway, as an increased amount of input power does not result in much shifting of the a* and b* values before immersion; the next result shows that the a* and b* values shift after immersion and that the color space serves the different locations of the L*-, a*-, and b* coordinates (Figs. S1–S3).

Figure 5a shows a reduction in the a* value, an increase in the L* value, and a decrease in the b* value after the immersion of the yellow printed paper. The shift in the L value from 20 kV is greater than that of untreated paper (0 kV), indicating increased brightness. Following plasma exposure, the cellulose fiber surface is notably stimulated, leading to the formation of new functional groups that impede the deactivation of pigment molecules, making them potentially difficult to deink^41^. However, our findings indicate a distinct behavior, as yellow ink appears to be easily removed after plasma exposure, during which new functional groups are formed.

The shift in the L* value from 16 kV (ΔL = 20.80) was greater than the shift in 0 kV or untreated paper (ΔL = 20.11), indicating that a change in brightness (L*) was achieved by blue dyes (Fig. 5b). A shift in the a* value and a decrease in the b* value after the immersion of the blue-printed paper were also observed.

Figure 5c depicts a decrease in the a* value and increases in the L* and b* values after immersion in red printed paper. The shift in the L* value from 20 kV was greater than that of the untreated paper (0 kV), indicating that a change in brightness occurred.

**Figure 6:**
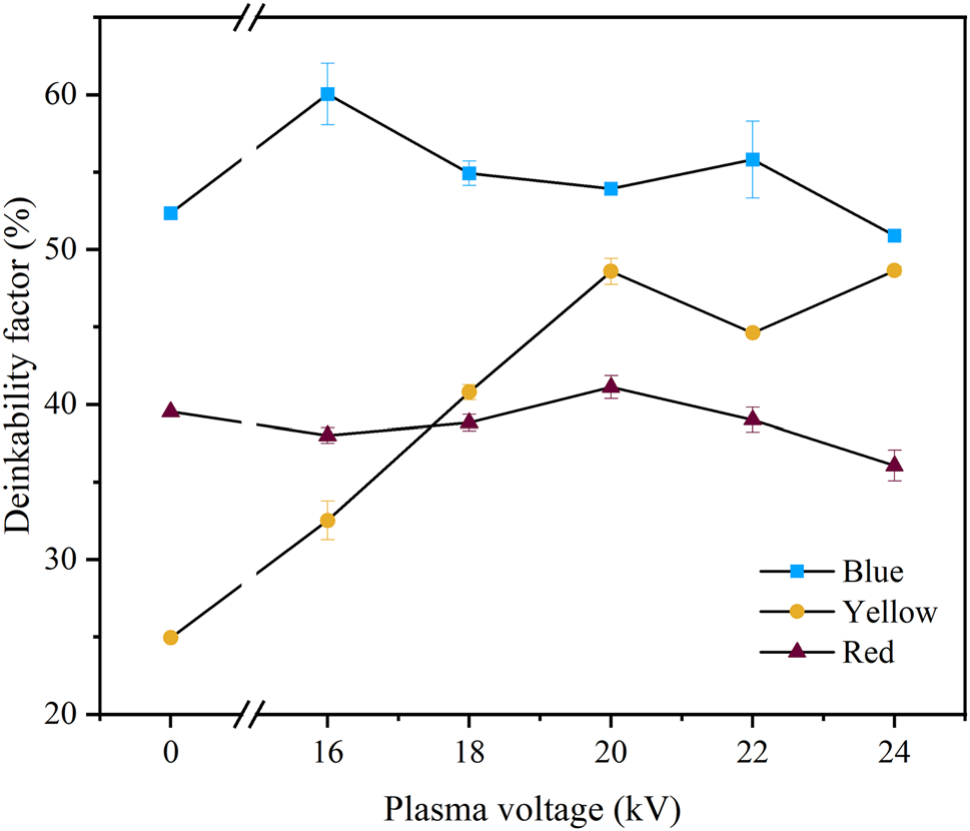
DEM_Lab_ was determined using a plasma exposure time of 10 min and an immersion time of 1 h.

The results in Fig. 6 demonstrate the deinkability performance calculation from the exact L*-a*-b* coordinates for all the experimental results of the 1st batch, where the plasma exposure time was 10 min on the 36 mm gap to the printed papers, tuned with a V_a_ of 16, 18, 20, 22, or 24 kV. A V_a_ of 0 kV represents the reference control condition where plasma is not applied. A V_a_ of 20 kV resulted in a maximum DEM_Lab_ of 48.58% on yellow paper, 16 kV yielded 60.04% on blue paper, and 20 kV yielded 41.11% on red printed paper. This result indicated that the plasma voltage played a significant role in increasing the removal indices of all these colors. This phenomenon may occur because polyelectrolytes are used in the paper industry to increase flocculation^42^. This agent can interact with plasma since coulomb interactions have occurred. Charged and noncharged polymers may lead to changes in ionic bonding, thus driving the dissolution of dyes^43^. Another plausible reason for this phenomenon is that an increased voltage can generate new functional groups on cellulose and lignocellulose surfaces that can modify their structure and chemical composition, facilitating the de-inking process via air plasma, and is proven to be an unconventional pretreatment method^44^.

### Performance of plasma exposure time in DEM_Lab_ and the physicochemical properties

**Figure 7:**
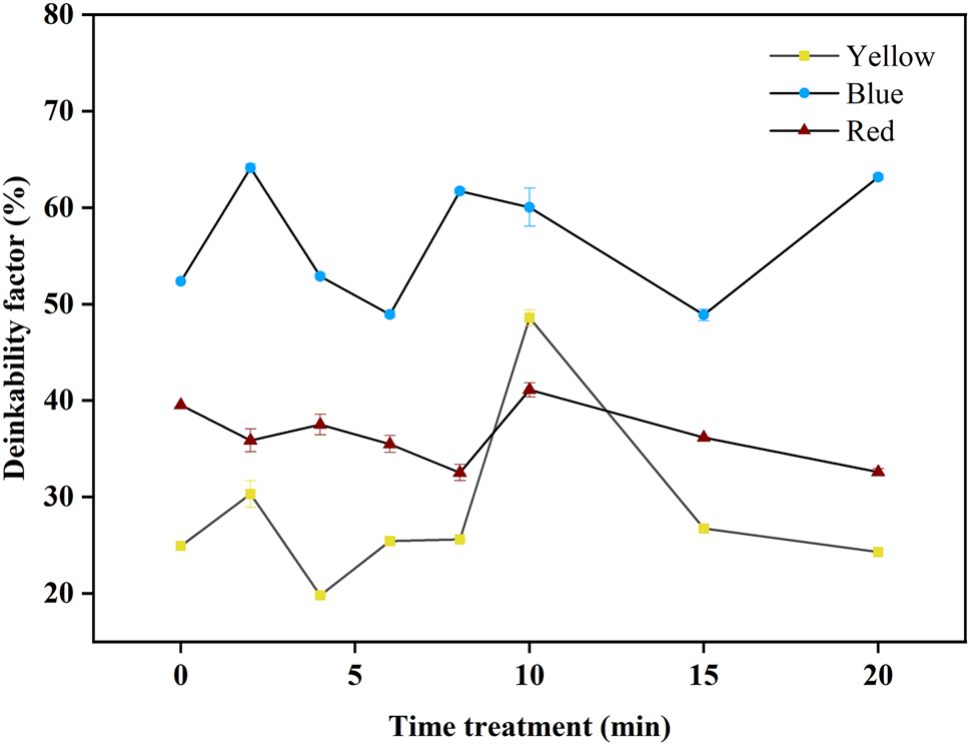
Summary of DEM_Lab_ values versus plasma exposure time using V_a_ values of 20, 16, and 20 kV for yellow, blue, and red printed paper, respectively.

To understand the time commitment, paper de-inking would need to be carried out over different exposure times (Fig. 7). According to the **DEM**_**Lab**_ results, the impact of time varied. In the first 2 min of treatment, the blue paper reached its optimal value (64.11%), while at longer times, the value decreased (4–6 min) (52.88–48.93%) and subsequently increased after 8 min (61.69%). This sequence was repeated when the plasma exposure time was increased to 20 min. The result decreased at 15 min (48.87%), and increased at 20 min (63.16%).

Although the blue ink achieved greater deinkability (64.11%) than the yellow and red ink, the fluctuating trend in the de-inking process of the blue pigment derived from copper phthalocyanine can be correlated with the molecular behavior observed during plasma thermal deposition. Previous reports have investigated the interactions of copper (II) phthalocyanine (blue dye) with H_2_O vapor/O_2_^45^. In this work, the plasma was air plasma, and therefore, oxygen and nitrogen radicals were generated. The thermal deposition process aligns copper phthalocyanine molecules through weak van der Waals and π-π interactions, promoting electron delocalization and facilitating the formation of longer molecular chains and elongated crystallites. The growth of the crystalline domain appears to be associated with the surface porosity and roughness. These correlations suggest that molecular alignment during deposition may influence the adhesion of ink to substrates, affecting its acceptance of the de-inking process. Understanding these molecular dynamics provides insights for optimizing the de-inking process, potentially mitigating fluctuations by adjusting thermal deposition conditions or other relevant parameters.

For the red ink, the deinkability values for untreated and treated red paper, when exposed to treatment times ranging from 0 to 20 min, were calculated to be 39.53, 35.87, 37.52, 35.49%, 32.54, 41.11, 36.18, and 32.59%. Notably, within the first 10 min of treatment, the red paper achieved an optimal deinkability value of 41.11%. However, a lower percentage of deinkability was observed compared to the corresponding trend in the case of blue ink. This result indicated that 2-Naphthalenol pigments, which are red dye constituents, have electrochemically stable derivatives^46^. This divergence in trends suggests that the electrochemically stable derivatives of 2-Naphthalenol pigments play a distinctive role in the de-inking process, emphasizing the complexity of ink and paper interactions.

The yellow ink results revealed deinkability values of 24.94, 30.31, 19.81, 25.43, 25.61, 48.58, 26.73, and 24.30% at 0 (untreated) to 20 min of treatment. After 10 min of treatment, the yellow paper yield reached its optimal value (48.58%). The yellow ink showed a lower deinkability than the blue ink but a higher deinkability than the red ink.

**Figure 8:**
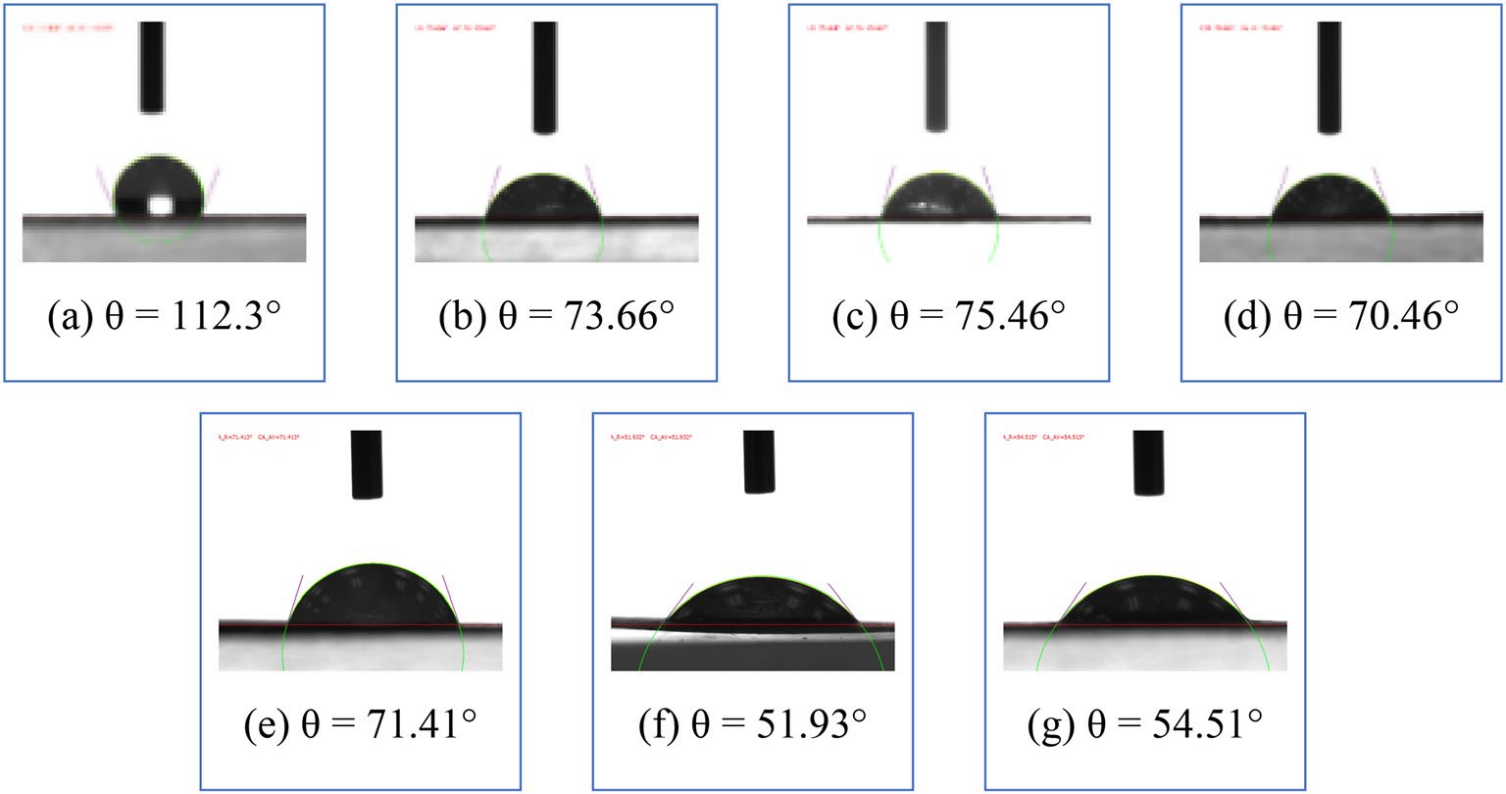
Water contact angle images of untreated samples for (**a**) white paper, (**b**) yellow printed paper on the front, (**c**) blue printed paper on the front, and (**d**) red printed paper on the front and plasma-treated samples for (**e**) yellow printed paper at 10 min on the front, (**f**) blue printed paper at 2 min on the front, and (**g**) red printed paper at 10 min on the front.

In addition to deterioration, physiochemical properties such as hydrophilicity are of interest since they help researchers understand ink–water interactions as a result of plasma treatment. The contact angle measurements in water for the different samples are recorded in Fig. 8. The contact angle on white paper can be seen in Fig. 8a. Commonly, lignocellulosic fibers are intrinsically hydrophilic, causing low contact angles with water. However, the unexpected contact angle shown in Fig. 8a of 112.3° indicates that the industrial-grade writing paper can be hydrophobic due to its contained coupling agent^47^.

After printing with different colors, the contact angle decreases. Previous work showed that the inhomogeneous structures of paper can lead to variations in ink penetration, which was found to be in the range of 43.25–68.62 µm^48^. Thus, during plasma treatment, the front side of the yellow surface shifted from 73.66° to 71.41° in 10 min (Fig. 8b,e). Moreover, the blue surface shifted from 75.46° to 51.93° in 2 min (Fig. 8c,f), and the red surface shifted from 70.46° to 54.51° in 10 min (Fig. 8d,g).

**Figure 9:**
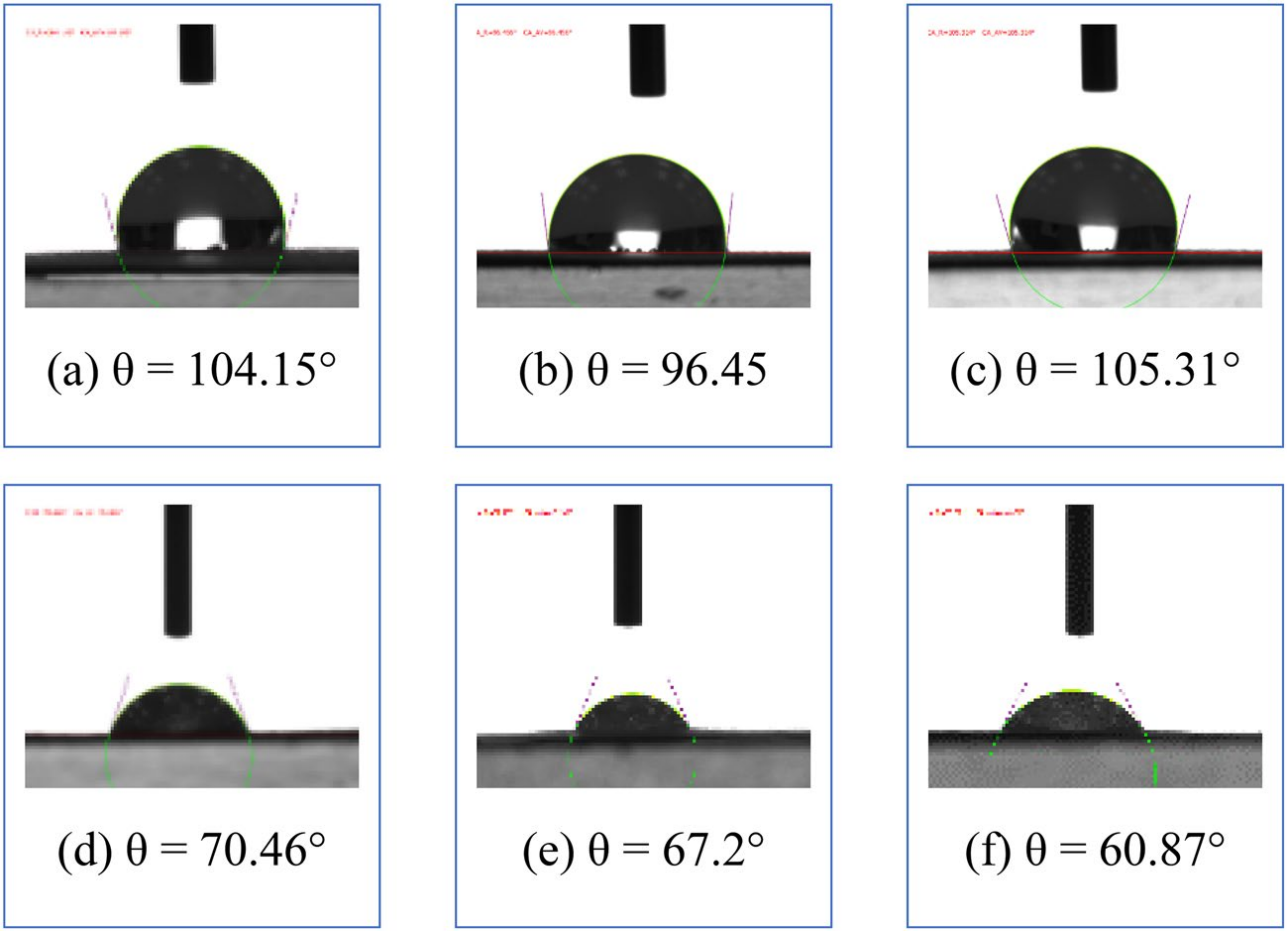
Images of water contact angle measurements on untreated samples for (**a**) yellow printed paper on the back side, (**b**) blue printed paper on the back side, and (**c**) red printed paper on the back side and plasma-treated samples for (**d**) yellow printed paper at 10 min on the back side, (**e**) blue printed paper at 2 min on the back side, and (**f**) red printed paper at 10 min on the back side.

The plasma treatment of the paper causes a decrease in the contact angle (θ) of the backside of the printed papers, as illustrated in Fig. 9. For example, when the yellow backside surface shifts from 104.15° to 70.46°, the blue backside surface shifts from 96.45° to 67.2°, and the red backside surface shifts from 105.31° to 60.87°, yielding hydrophilicity, which is a good indication of ink diffusing into water during the de-inking process. This finding indicates that the corona discharge plasma penetrates the front surface and deep into the back side of the paper, increasing the hydrophilicity of the paper. Michlíček et al.^49^ found that microdischarges created from corona plasma deepen the penetration because the Debye length is approximately 2 mm $\times {10}^{-4}$. It was concluded that between microdischarge events, electrons strongly collide with neutral species due to the high density of electrons. At that density, ionization processes are the most significant mechanism of energy transfer^50^, suggesting alteration of the chemical structure of the paper hydrophilicity.

**Figure 10:**
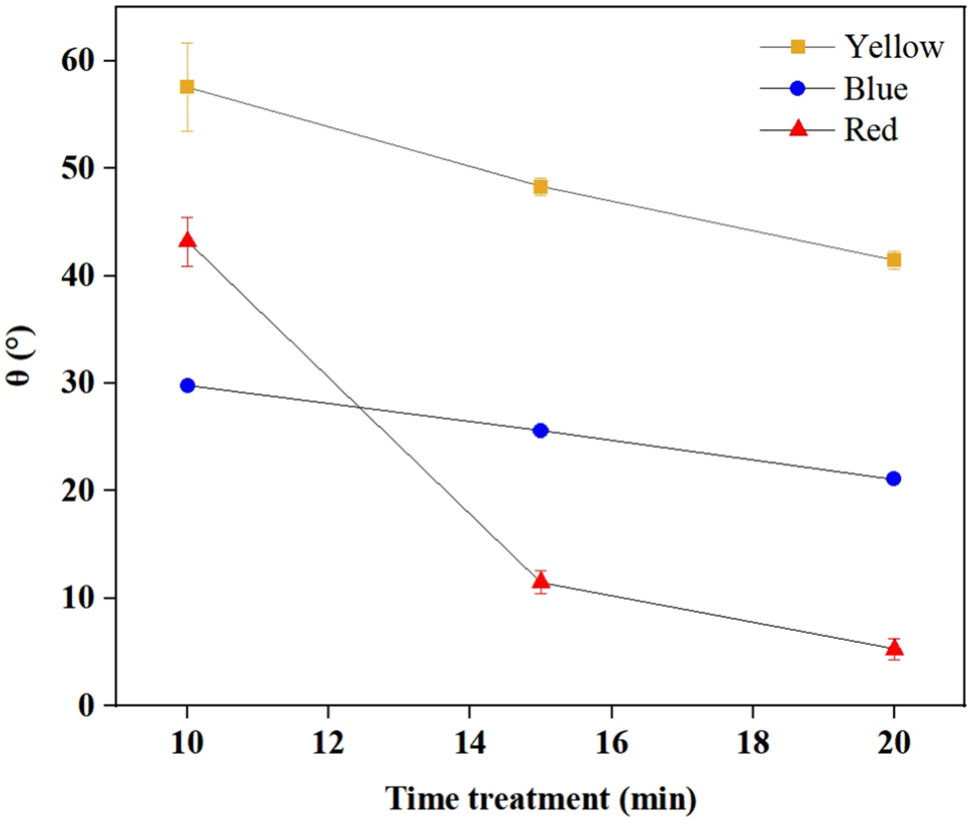
Results of contact angle measurements on the front side at longer plasma treatment times for yellow, blue, and red printed paper.

As presented in Fig. 7, prolonging the plasma exposure time (beyond 10 min) did not increase the deinkability, as illustrated by the decreasing results, suggesting that the exposure time does not impact the result of deinkability ink materials, except for the 20 min treatment of the blue printed paper. This phenomenon indicates that an apparent saturation point of the RONS occurs with extended plasma exposure duration^51^. Notably, the relatively long treatment time undergoes tuning, suggesting an increased level of hydrophilicity due to plasma induction, increasing the paper surface polarity and surface roughness. This event is observed in the contact angle results, as shown in Fig. 10.

Equation (3) calculates the adhesion energy between a droplet of water and the surface, referred to as Young–Duprès^33^:

3 $${W}_{T}^{A}= {\gamma }_{L}^{T}(1+\text{cos}{\theta }_{w/pap})$$

where ${\text{W}}_{\text{T}}^{\text{A}}$ is the adhesion energy of the liquid to a solid, ${\upgamma }_{\text{L}}^{\text{T}}$ is the surface tension of the liquid (water in the present case, i.e., 72.8 mJ/m^2^ at 20 °C), and ${\uptheta }_{\text{w}/\text{pap}}$ is the water contact angle on the paper surface. These results are reported in Table 2.

**Table 2:** Adhesive energy before and after plasma treatment.

${W}_{T}^{A}$ (mJ/m^2^)^a^	${W}_{T}^{A}$ (mJ/m^2^)^b^			Exposure time (min)	${W}_{T}^{A}$ (mJ/m^2^)^c^		
	Yellow	Blue	Red		Yellow	Blue	Red
48				10	111.8	136.0	125.9
	93.3	91.1	97.7	15	121.2	145.5	144.1
				20	127.4	150.3	145.4

^a^White paper, ^b^after printing, ^c^after printing and plasma treatment.

Before color printing, the adhesion energy (${{\varvec{W}}}_{{\varvec{T}}}^{{\varvec{A}}}$) of the white paper was 48 mJ/m^2^ whereas, after printing, it was approximately double in all the samples studied here (93.3, 91.1, and 97.7 mJ/m^2^ for yellow, blue, and red printed papers, respectively). After plasma treatment, the value was approximately three times greater, i.e., 150.3 mJ/m^2^, in blue printed paper. This result confirmed that the paper surface underwent chemical changes. Therefore, reactive polar groups are introduced and the surface roughness is increased by prolonging the exposure time^33^.

### Performance of plasma gap sizes on DEM_Lab_

**Figure 11:**
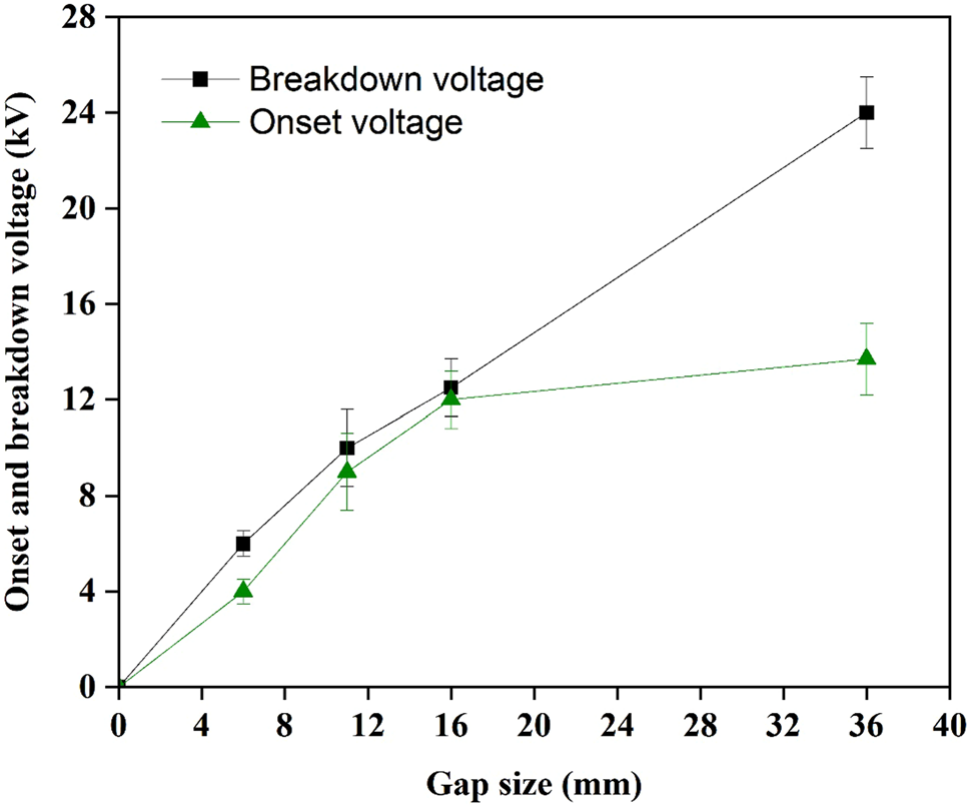
Effect of the gap size between the paper and needle tip on the onset and breakdown voltage.

The color-printed paper was exposed to plasma with different gap sizes of 6, 11, 16, and 36 mm. This gap was measured between the tip of the needle and the surface of the targeted paper. The breakdown voltages for all the colors are the same because the thicknesses of the papers are identical. Therefore, the effect of the gap size on the breakdown voltage of the printed paper is demonstrated in Fig. 11.

A common feature of corona discharge is a considerable gap accompanied by a relatively high breakdown voltage. Pushing the applied voltage to a value higher than the breakdown threshold results in an unstable or disappearing corona discharge with the generation of continuous sparks, which is a disadvantage in the present investigation. This breakdown occurs when the applied electric field is higher than the critical value (E_cr_/N or the threshold of electric field for the onset corona voltage^52^), and streamer propagation starts to occur when the electron avalanche growth reaches its critical size^30^. In agreement with Wozniak et al.^53^, the observed gap exhibited similar behavior according to the Meek’s criteria^54^. This phenomenon shows that a higher breakdown voltage accompanies an enormous gap, as indicated for the 36 mm gap (the maximum gap available for this device).

**Figure 12:**
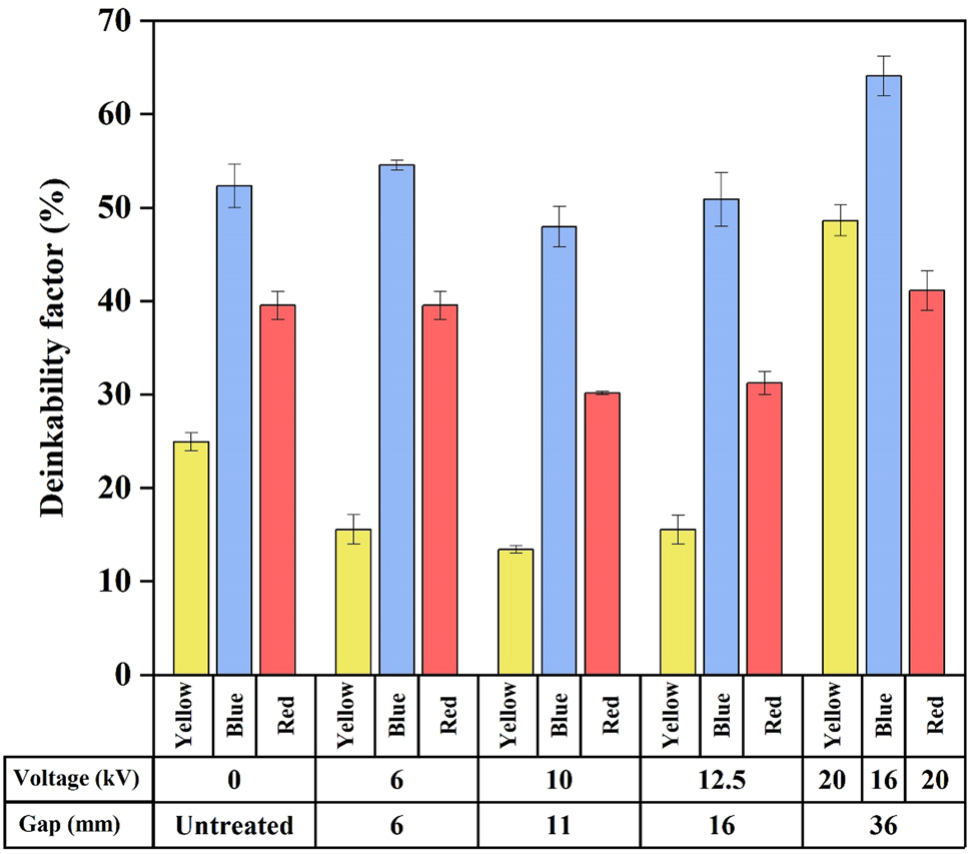
Effect of electrode gap on the deinkability factor.

A suitable electrode gap is critical because it affects the electric field and influences the plasma performance. The effect of the gap size on the DEM_Lab_ for different printed colors is demonstrated in Fig. 12. According to the results of this study, increasing the gap until 16 mm did not influence the DEM_Lab_ of all paper; it is hypothesized that a relatively short lifetime of radical species probably limits the participation in chemical reactions on the paper surface when having an unsuitable gap due to a narrow working window between onset voltage and breakdown voltage (Fig. 11). These species can also collide with species near the electrode head space before eventually reaching the surface to breakdown and de-ink the paper. The loss probability for radicals at the surface that is taken into account by Hori report^55^ also explains that this probability varies greatly depending on the interactions of the radical with paper surfaces. Typically, when the electrode gap is relatively short (6–16 mm), faster ion mobility occurs. However, less interaction time affects processes between ion and paper surface^56^. Thus, the high-energy electrons and active particles from atomic nitrogen, oxygen, and other gases contained in the air cannot take full effect, leading to an inefficient process and reducing the deinkability of all paper. Therefore, it is difficult to detach the ink into water during the immersion process. These observations suggest that the gap sizes (6–16 mm) of plasma cannot affect the residual paper ink significantly^57^. However, the 36 mm gap resulted in optimal deinkability due to the working window of plasma voltage appearing to fulfill a suitable gap criterion (see Fig. 11), suggesting that this gap provided the highest number of radicals to react with paper surfaces.

### UV–Vis, ATR-FTIR and DART-MS evaluation

**Figure 13:**
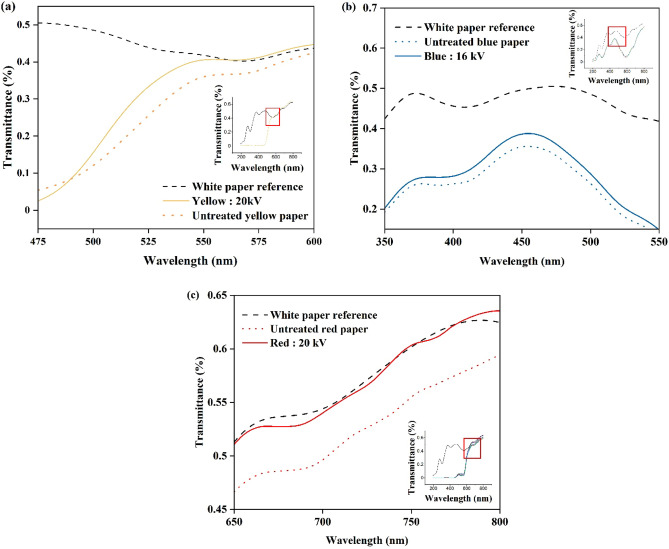
UV–Vis measurement on printed paper over gap length for (**a**) yellow, (**b**) blue, and (**c**) red papers.

To further confirm the dye decomposition mechanism, transmittance measurements of the yellow, blue, and red printed papers were conducted to test the residual ink on the printed paper with a UV‒Vis spectrometer. Figure 13a–c show the specified wavelength range of each class of dye chemical wavelength^58^. It was found that higher transmittance values were observed for the plasma-treated samples compared with the nontreated ones after immersion in water, under the optimal conditions of 20 kV (yellow and red) and 16 kV (blue) plasma discharge voltages, 10 min plasma exposure time, 36 mm plasma gap, and 1 h immersion time.

For the yellow-printed paper (Fig. 13a), part of the wavelength peak (λ = 550–600 nm,^58^) was found to reach a similar value to that of the white reference paper, signifying that the remaining residual ink was destroyed through plasma exposure. In contrast, the results for the blue-printed paper (Fig. 13b) showed that slightly higher transmittance values were observed in the plasma-treated samples than in the nontreated one (λ = 450–550 nm^58^,), but the value never reached that of the white reference paper, signifying that the concentration of blue dye is reduced compared to untreated one, although there was some remaining residual ink not destroyed through plasma exposure. Figure 13c indicates that the transmittance values (λ = 650–700 nm,^58^) of the red plasma-treated samples were significantly higher than that of the nontreated one, and even reached that of the white reference paper. It suggests that the remaining residual ink was remarkably destroyed through plasma exposure.

**Figure 14:**
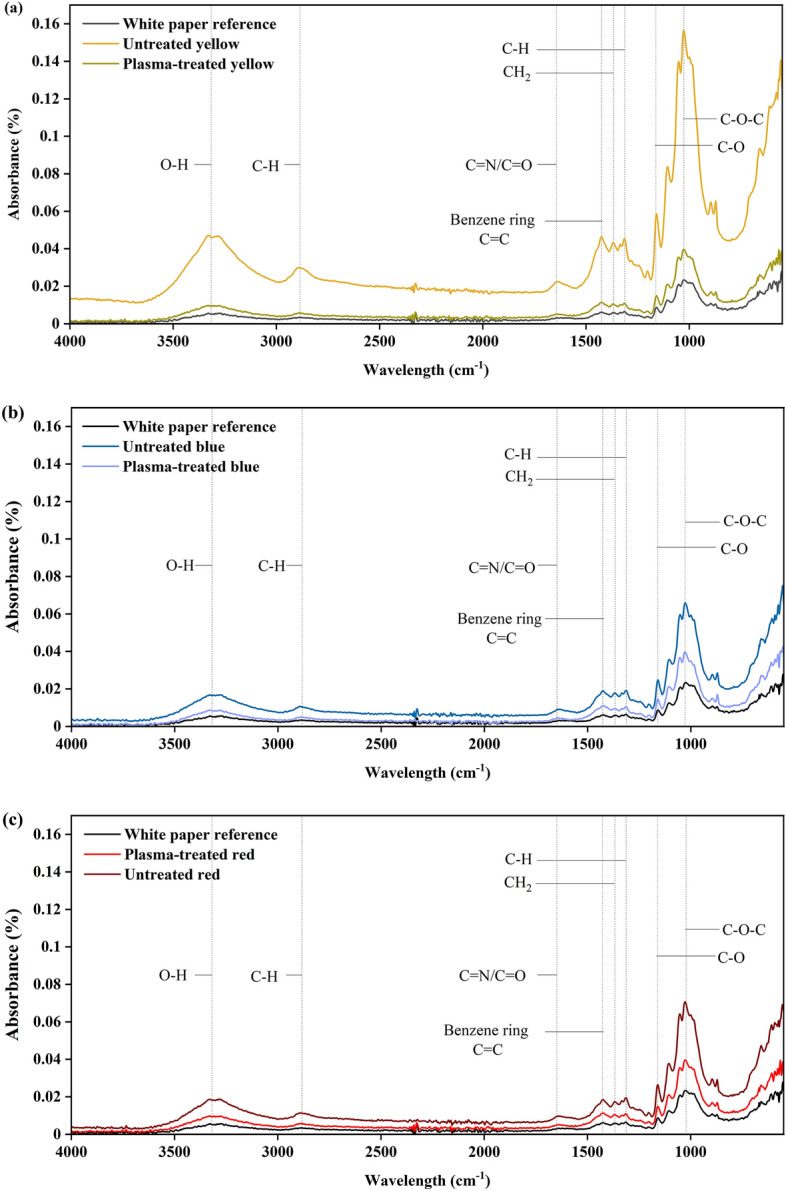
ATR-FTIR measurement on printed paper over gap length for (**a**) yellow, (**b**) blue, and (**c**) red papers.

To confirm the impact of corona discharge plasma on paper chemistry, ATR-FTIR measurements are conducted. Figure 14a–c compare all plasma treated and non-treated articles.

The absorbance peaks in all samples were mostly relate to cellulose as the paper constituent material. As described in the literature, OH, CH, C–O, and C–O–C bonds (3300, 2890, 1370, and 1315 cm^−1^) belong to the properties of cellulose ^57,59,60^. The broadening peak at 1640 and 1427 cm^−1^ with a low intensity may be attributed to the presence of C=N bonds and C=C benzene rings frame, especially after printing yellow, blue, and red ink on white paper (which lacked these peaks). The C=N bonds might overlap with other bonds such as C=O or NH. The study indicated that after plasma treatment, the intensity of C=N bonds of yellow, blue, and red case approached the reference of the white paper (Fig. 14a–c). This suggests that plasma treatment potentially degrades dye molecules present in the ink compounds.

**Figure 15:**
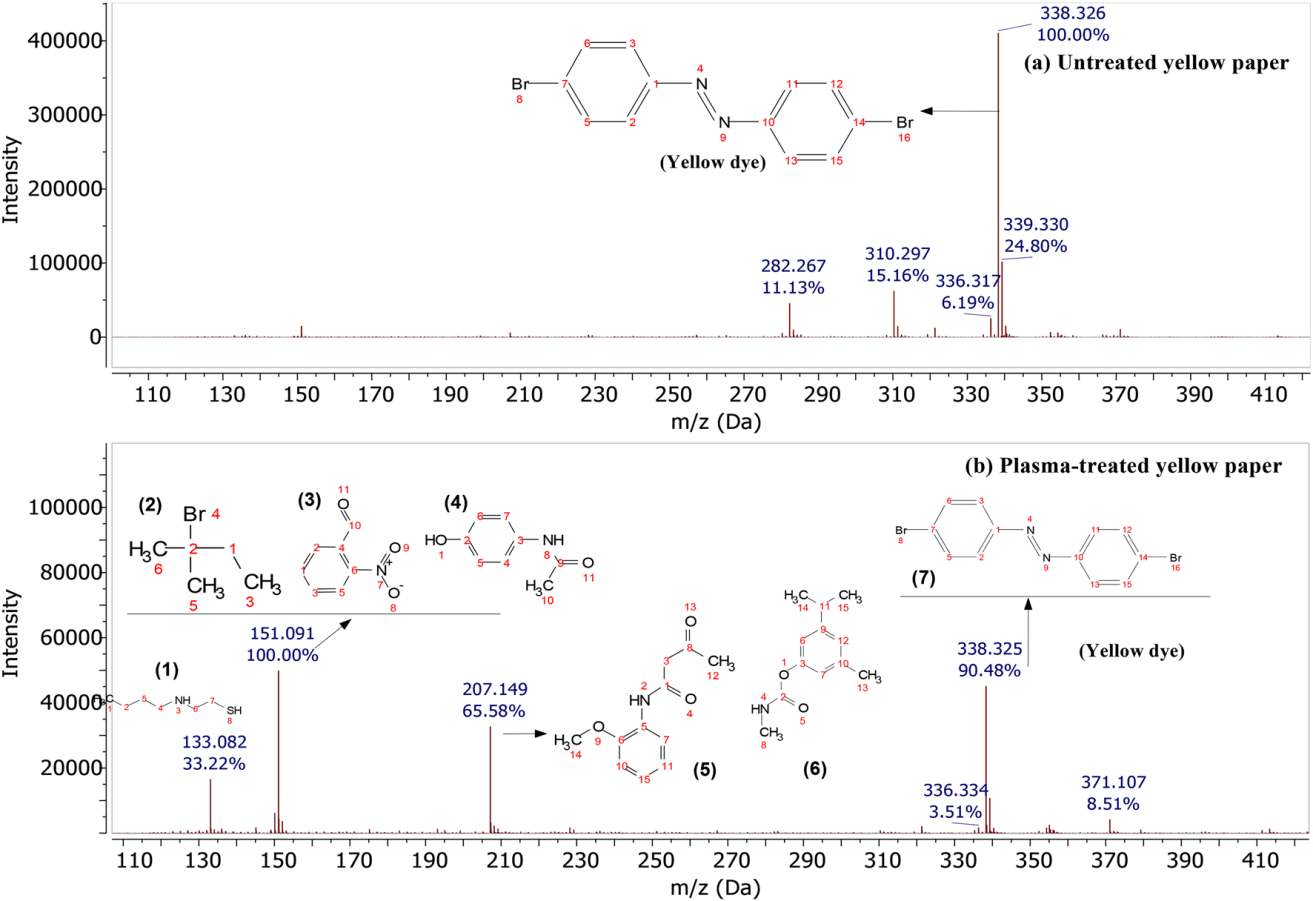
DART-mass spectra of yellow case (**a**) untreated and (**b**) plasma-treated yellow paper.

Figure 15 shows the DART-mass spectra for (a) untreated yellow paper and (b) plasma treated yellow paper. The spectra show typical peaks of their characteristic ink after printing. The distinctive peaks observed at mass-to-charge ratio (m/z) of 336, 338, and 339 signified the presence of the yellow dye/diazene,bis(4-bromophenyl) compound in the untreated yellow paper (Fig. 15a). Both eicosane and docosane peaks were also identified at m/z 282 and 310 corresponding to facilitating the emulsification of water and oil phases^61^. They were identified within the yellow dye component of ink-jet printing ink, suggesting their favourable properties such as high latent heat capacity and chemical stability which are commonly needed in the ink industry^62^. Ink-jet inks often comprise colloidal particles, encompassing pigment particles, polymers, and occasionally emulsion droplets, which interact through collisions. The presence of these colloidal particles causes them to collide with each other in printing process. After plasma was introduced (Fig. 15b), the relative abundance or intensity of yellow dye at m/z of 338 was decreased from 100 to 90.48%. It was found that the 100% relative abundance was formed at m/z of 151, followed by other new fragments at m/z of 133 (33.22%), and at m/z of 207 (65.58%). Figure 15b also depicted 3 possible structures of compounds predicted by mNova software at m/z of 151 (see Fig. 15b 2,3 and 4), while 2 possible structures were matched at m/z of 207 (see Fig. 15b 5 and 6). In the process of plasma treatment, the azo N = N bond was initially targeted by highly reactive species and appeared to produce a reasonable structure compound of 5-amino-2-hydroxybenzoic acid (4) with m/z of 151^63^. Subsequent cleavage of the benzene ring led to the generation of 2-(butylamino) ethanethiol (1) with m/z of 133, as well as N-(2-methoxyphenyl) butanamide/methylcarbamate (5,6) with m/z of 207. A mixture of small-molecule organic acids may result in further breakdown of these compounds.

**Figure 16:**
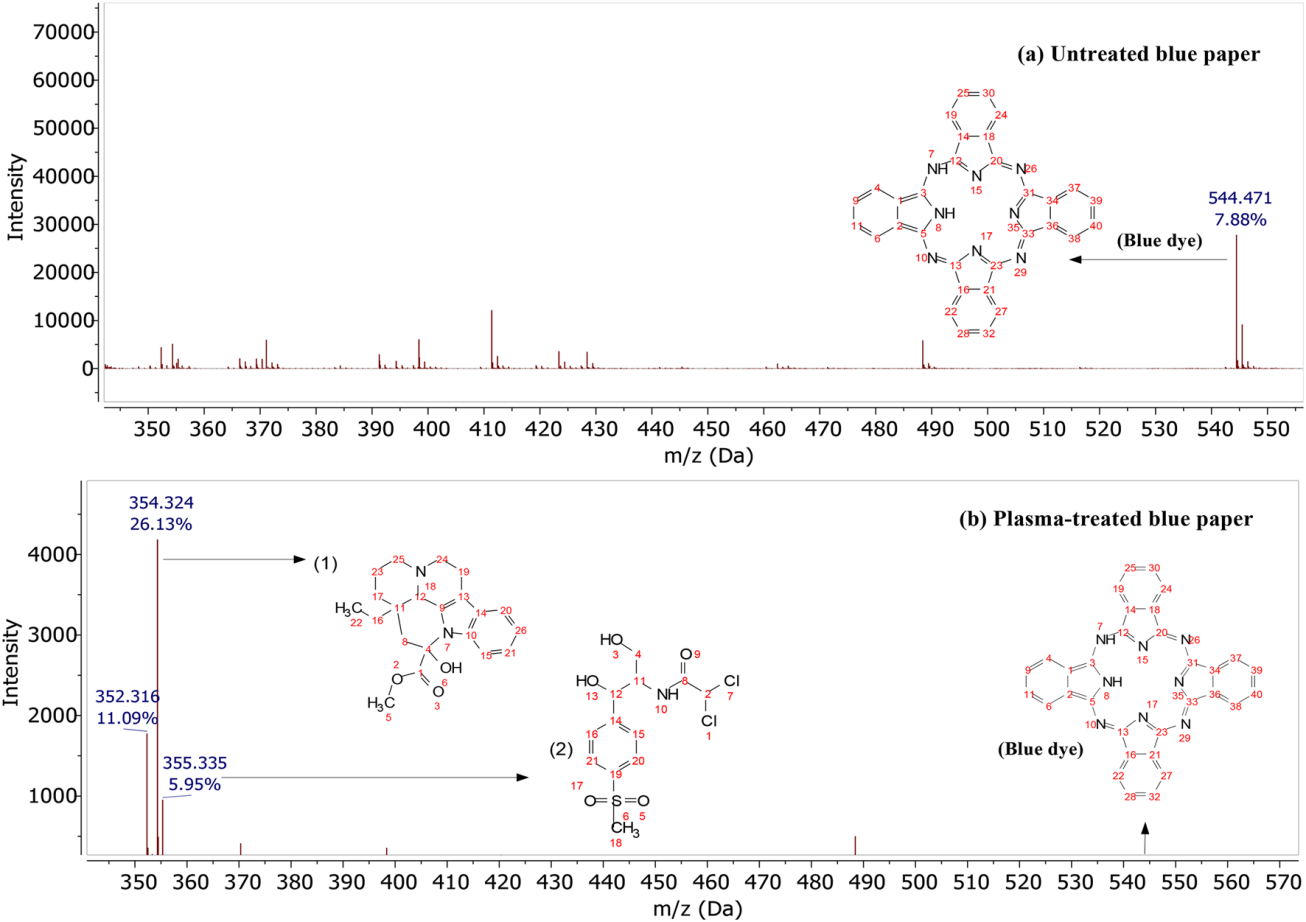
DART-mass spectra of blue case (**a**) untreated and (**b**) plasma-treated blue paper.

The degradation of the blue dye was observed, and the DART-MS spectra depicted in Fig. 16a,b. The presence of phthalocyanine was identified at m/z of 544 in Fig. 16a. After plasma treatment, the relative abundance of m/z of 544 significantly decreased from 7.88% to zero (Fig. 16b). Other identified fragments were observed at m/z of 352–355. The highest relative abundance of these fragments was predicted to be vincamine (1) which consist of: 11.09% at m/z of 352 (C_21_H_26_N_2_O_3_ loss of H^+^) and 26.13% at m/z of 354 (C_21_H_26_N_2_O_3_ adduct/loss of e^−^)), followed by 5.95% of thiamphenicol (2) at m/z of 355. In this process, cleavage of the benzene ring may initially be targeted by highly reactive species, leading to the generation of both fragments. Phthalocyanine can have an average of 0.25 to less than 1.5 of the SO_2_NH-alkyl-OH groups, suggesting that thiamphenicol at m/z of 355 has a sulfur atom^64^. In summary, these results indicate that corona plasma de-inking could effectively degrade blue dyes.

**Figure 17:**
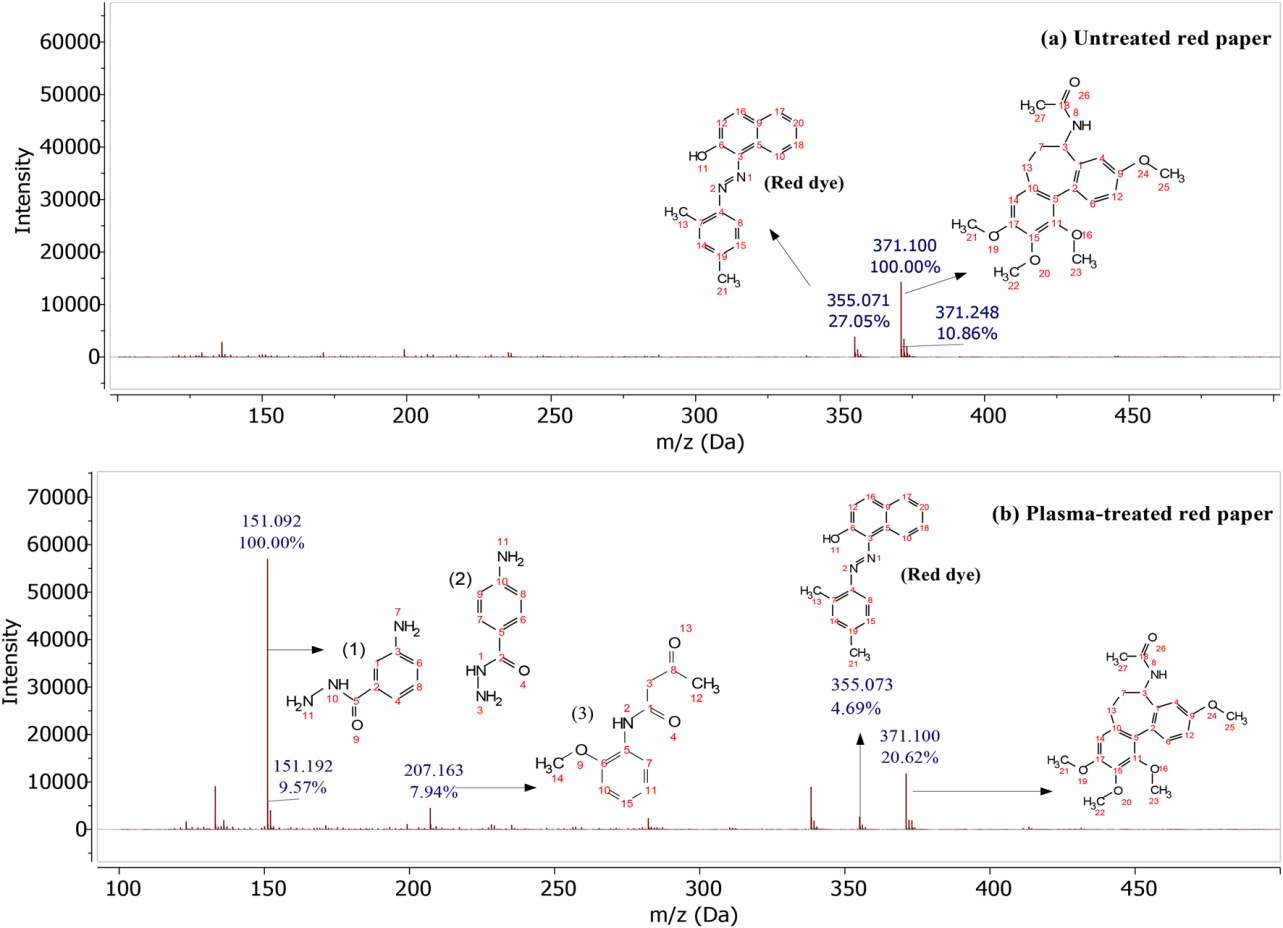
DART-mass spectra of red case (**a**) untreated and (**b**) plasma-treated red paper.

Figure 17 illustrates the DART-mass spectra for the red case. The 2-Naphthalenol peak at m/z of 355 was detected in Fig. 17a while the other constituent at m/z of 371 was present with the most relative abundance of N-Acetylcolchinol methyl ether. After plasma treatment (Fig. 17b), the relative abundance of 2-Naphthalenol became 4.96% and N-Acetylcolchinol methyl ether was 20.62%. Besides, other fragments were found at m/z of 151 and 207, suggesting the radicals may degrade red dyes into (1) 4-Aminobenzoic acid, (2) 3-Aminobenzhydrazide, and (3) N-(2-methoxyphenyl)butanamide. The most relative abundance at m/z of 151 may generates 2 types of fragment compounds such as 3-Aminobenzhydrazide or 4-Aminobenzoic acid due to the mass target similarity of both compounds. Panizza et al.^65^ found that a low concentration of benzoic acid was generated during the study of the electrochemical oxidation of 2-naphthalenol. The present investigation revealed that 3-Aminobenzhydrazide or 4-Aminobenzoic acid had 100% relative abundance. Indication of the degradation pathway of 2-naphthalenol occurred by forming an amino group (NH_2_) at the para position of the benzene ring, suggesting that the amino group can be formed from the interaction between RONS (reactive oxygen and nitrogen species) from air plasma with precursors containing nitrogen functional groups (azo). In the process of plasma treatment, the azo N=N bond was initially targeted by highly reactive species rather than the cleavage of the benzene ring.

To summarize the possible mechanisms of the de-inking, it can be considered that plasma radicals can initiate oxidation reactions through the decomposition of the dye molecules into smaller fragments. The hydroxyl radicals (⋅OH) or nitrogen radicals (N⋅) may also contribute to the oxidative cleavage of nitrogen-containing functional group (azo N=N), resulting in N bond separation process within the dye molecule structure.

### SEM and tensile tests for determining fiber morphology

**Figure 18:**
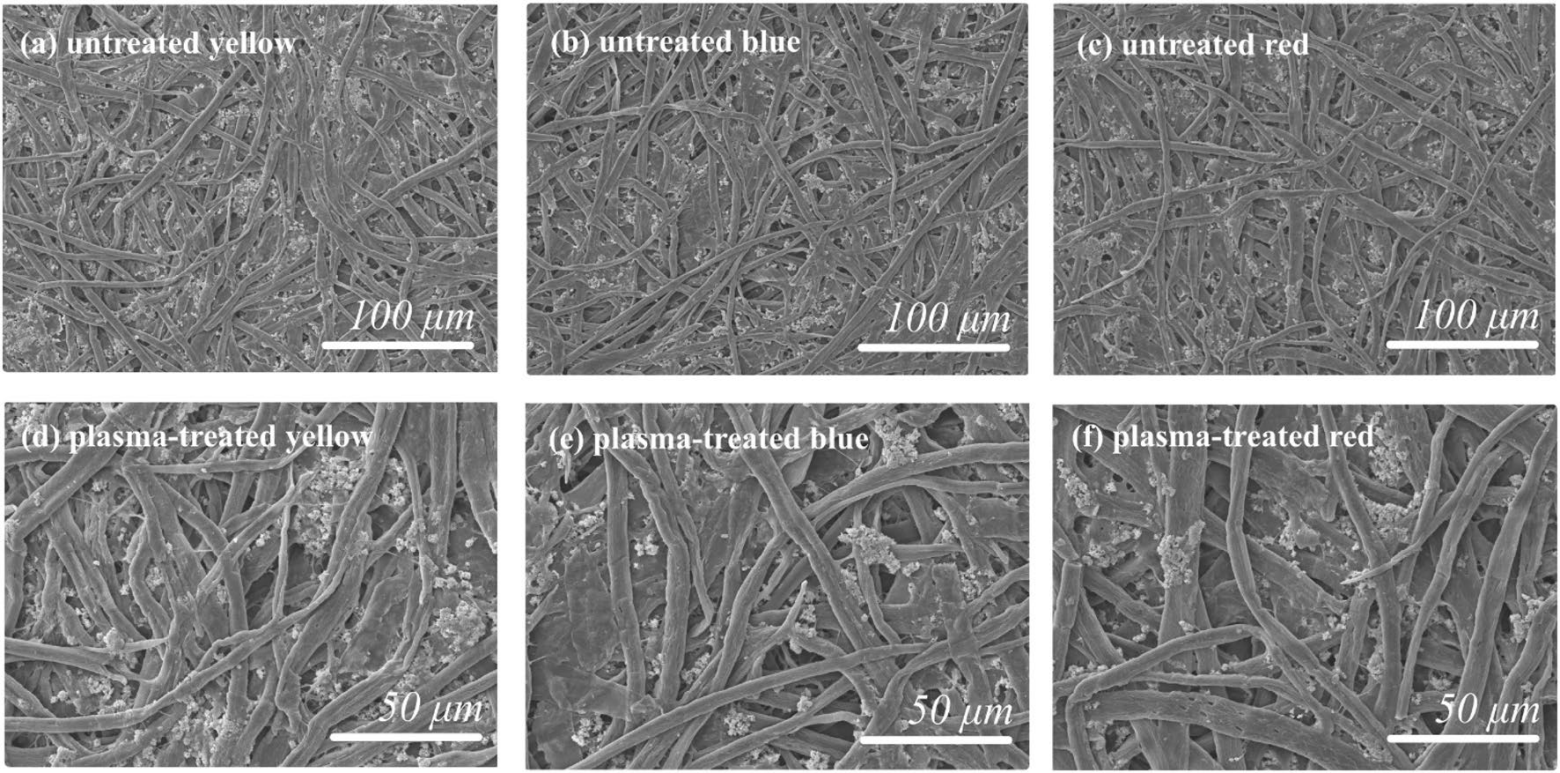
SEM micrographs before-after 15 min of plasma treatment.

SEM was used to observe the surface of the plasma-treated printed paper. After 15 min of plasma processing (Fig. 18a–f), the morphology of the paper fibers upon corona discharge plasma treatment were not damaged. Although the oxidation of surface fibers occurs because oxygen-containing species are present in the surrounding environment of the air plasma source and lead to the formation of certain functional groups, such as –OH or –COOH, no noticeable differences can be observed in the plasma-treated papers^57^.

From a physical perspective, the mechanical properties of the paper were assessed using a tensile test, and the results are presented in Table 3.

**Table 3:** The mechanical properties of the paper were evaluated via tensile tests.

Condition	Tensile strength (N/mm^2^)	Breaking factor (N/mm)
Untreated white paper	5065 ± 487.44	8.81 ± 0.29
Untreated printed paper	2339 ± 190.98	4.07 ± 0.10
Plasma-treated printed paper	4593 ± 248.47	7.51 ± 0.25

A decrease in the tensile strength was observed between the untreated white paper and the untreated printed paper (5065–2339 N/mm^2^). This decrease seems to be attributed to excess moisture, primarily from the solvents and pigments in the inks after the printing process. An excess amount of moisture is attributed to the presence of a water-based polymer in the pigment ink, which plays a crucial role in printing fluency in inkjet-printing ink^66^. This excess moisture may weaken the cellulose fiber–fiber bonds at the surface and subsequently reduce the tensile strength of the material^67^. Additionally, the bonding strength of cellulose fibers in paper is attributed to hydrogen bonding, the bonding mechanism between fibers, which is widely regarded as the most significant factor influencing paper properties^68^. Weakening of the hydrogen bonds or the cellulose fiber–fiber bonds results in the loss of fiber swelling and wet flexibility^69^. It may be concluded that this reduction is an acceptable process when exploring the physical performance of printed paper.

However, the tensile strength of the plasma-treated printed paper closely approached that of the untreated white paper (4593–5065 N/mm^2^). A less than 10% decrease in tensile strength due to the 2 min corona plasma treatment was in line with the findings of a study by Putra et al.^70^. Another study reported a tensile strength reduction of approximately 14% with coir fibers after receiving a liquid plasma treatment^71^. This difference appears to be obtained because the addition of air plasma to the surface of natural fibers excites the oxygen functional group concentration^72^. In contrast, this reduction is relatively small compared to the tensile strength reduction of recycled paper in common recycling. For example, Jin et al.^68^ investigated the decrease in tensile strength in hand sheets prepared from several types of pulp via three recycling cycles. A reduction of approximately 14–18% was obtained after the first to the third recycling cycles. In addition, Li et al.^69^ predicted that approximately 10–40% of the decrease in the tensile strength of paper sheets would occur after four recycling cycles. It may be concluded that a relatively low effect is induced by corona plasma treatment during the de-inking process.

For the results of the breaking factor, the value for the untreated white paper was 8.81 ± 0.29 N/mm, the value for the untreated printed paper was 4.07 ± 0.10 N/mm, and the value for the plasma-treated printed paper was 7.51 ± 0.25 N/mm. These results give a similar indication pattern to the tensile strength records. In contrast to the findings of Mauchauffe et al.^57^, the force at the break for nontreated white paper was 4.65 ± 0.9 N, and that for plasma-treated white paper was 4.77 ± 0.3 N. Although the values are similar, the results are different from our findings because it is assumed that the positive, negative, or unchanging influence of plasma treatment on the tensile strength can depend on the treatment conditions since the plasma treatment promotes fiber–fiber adhesion by introducing polar, excited groups or a new polymer layer to establish strong covalent connections.

In all the papers, whiteness and yellowness were measured. These parameters of recycled paper are essential because the appearance of the paper directly affects the acceptability of the paper product standard by the International Organization for Standardization (ISO) 11,475. The blue and red paper samples initially had negative whiteness values that strongly increased with increasing treatment time (Table 4) and had the positive yellowness values that slightly similar before-after the treatments. In yellow paper, the whiteness value was positive and only slightly increased by the treatment, while the yellowness value was fluctuated in negative value and increased to a relatively high negative value after 15 min.

**Table 4:** Whiteness and yellowness of yellow, blue, and red printed papers.

Condition	Whiteness (ISO 11475)			Yellowness (American society for testing and materials (ASTM) E313-10)		
Treatment time (min)	Yellow	Blue	Red	Yellow	Blue	Red
2	148.37	− 181.42	− 100.36	− 45.92	77.2	71.34
4	144.61	− 172.42	− 111.62	− 44.8	76.25	73.84
6	148.51	− 186.47	− 107.61	− 47.73	79.94	74.56
8	140.35	− 176.94	− 120.11	− 34.75	76.31	79.54
10	145.40	− 180.76	− 110.65	− 38.35	70	76.51
15	150.15	− 171.75	− 108.05	− 48	77.81	75.43
20	150.92	− 184.23	− 110.82	− 48.9	77.87	75.46

## Conclusion

The work presented here features a plasma-based de-inking process of inkjet printer ink on several printed papers. The DEM_Lab_ percentage of printed paper significantly depends on the plasma V_a_ and plasma exposure time. Concerning the role of the gap size, the varied parallel plates do not significantly impact the DEMLab.

In this work, the influence of the applied plasma voltage on the L*, a*, and b* values and on the DEMLab values was studied under the optimal conditions of 20 (yellow and red) and 16 (blue) kV plasma voltages, a 10 min plasma exposure time, a 36 mm plasma gap, and a 1 h immersion time. The highest DEM_Lab_ values of yellow, red, and blue printed papers increased from 24.93, 52.34, and 39.52% to 48.58, 60.04, and 41.11%, respectively.

The dependence of DEM_Lab_ and contact angles on plasma exposure time were evaluated and discussed. The investigation of various treatment times from 2 to 20 min revealed that prolonging the plasma exposure did not affect the rate of deinkability but decreased the contact angle of all the papers, probably because the chemical characteristics of the paper changed. The longer the treatment time was, the more polar and rougher the paper surface. In addition, the influence of various gaps was extensively studied, and the results are presented for all paper samples. From these results, it is evident that the discharge gap has no significant impact on the DEMLab of paper de-inking. However, a 36 mm distance between the two electrodes is the optimal distance, and a wider distance would cause a breakdown of the voltage. Plasma treatment might help the recycling process, especially due to the shift in the paper's physicochemical surface from hydrophobic to hydrophilic for better paper pulp results.

Finally, this study establishes a pioneering result in de-inking paper research supported by the use of corona discharge plasma under atmospheric pressure. For the first time, corona discharge plasma has been successfully applied to the de-inking process. New methods are needed to create green de-inking processes in the paper recycling sector. Therefore, a sustainable method for removing ink from paper without toxic reagents or hazardous waste is preferable. Moreover, the absence of chemical surfactants in this system results in less solid or liquid waste. The green de-inking process can be scaled by utilizing inexpensive and plentiful gaseous air mixtures while working under atmospheric pressure conditions without expensive vacuum components. Based on the above findings, collaboration between paper recycling industries and developers of plasma technologies can mitigate high power consumption and achieve significant economic and environmental benefits.

### Supplementary Information

Supplementary Information.

## Data Availability

The data that support the findings of this study are available upon reasonable request.

## Abbreviations

GHG: Green house gas
TSIC: Thailand standard industrial classification
DTPA: Diethylene triaminepentaacetic acid/pentetic acid
PhAcs: Active pharmaceutical compound
$${DEM}_{Lab}$$: Deinkability factor
DBD: Dielectric barrier discharge plasma
NTP: Nonthermal plasma
