# Supplementary material for: Corona discharge plasma for green de-inking of inkjet printer ink
Source: Sci Rep. 2024 Jun 6;14:13035. doi: 10.1038/s41598-024-63683-8 (PMC11156896; doi:10.1038/s41598-024-63683-8)
Supplement: Supplementary file 1 — Supplementary Information. [file 41598_2024_63683_MOESM1_ESM.docx]

**Supplementary Material**

Corona discharge plasma for green de-inking of inkjet printer ink

Ika Priyanti^a^, Doonyapong Wongsawaeng^a*^, Kanokwan Ngaosuwan^b^, Worapon Kiatkittipong^c^, Peter Hosemann^d^, Suttichai Assabumrungrat^e,f^

–––––––––

*^a^ Research Unit on Plasma Technology for High-Performance Materials Development, Department of Nuclear Engineering, Faculty of Engineering, Chulalongkorn University, Bangkok 10330, Thailand*

*^b^ Division of Chemical Engineering, Faculty of Engineering, Rajamangala University of Technology Krungthep, Bangkok 10120, Thailand*

*^c^ Department of Chemical Engineering, Faculty of Engineering and Industrial Technology, Silpakorn University, Nakhon Pathom 73000, Thailand*

*^d^ Department of Nuclear Engineering, Faculty of Engineering, University of California at Berkeley, 94720, U.S.A.*

*^e^ Center of Excellence in Catalysis and Catalytic Reaction Engineering, Department of Chemical Engineering, Faculty of Engineering, Chulalongkorn University, Bangkok 10330, Thailand*

*^f^ Bio-Circular-Green-economy Technology & Engineering Center (BCGeTEC), Faculty of Engineering, Chulalongkorn University, Bangkok, Thailand 10330*

**Table S1**. V-I signals of yellow printed paper.

| **No** | **DSO** | **HVDC**  **(kV)** | **Applied current (mA)** |
| --- | --- | --- | --- |
| **(a)** | 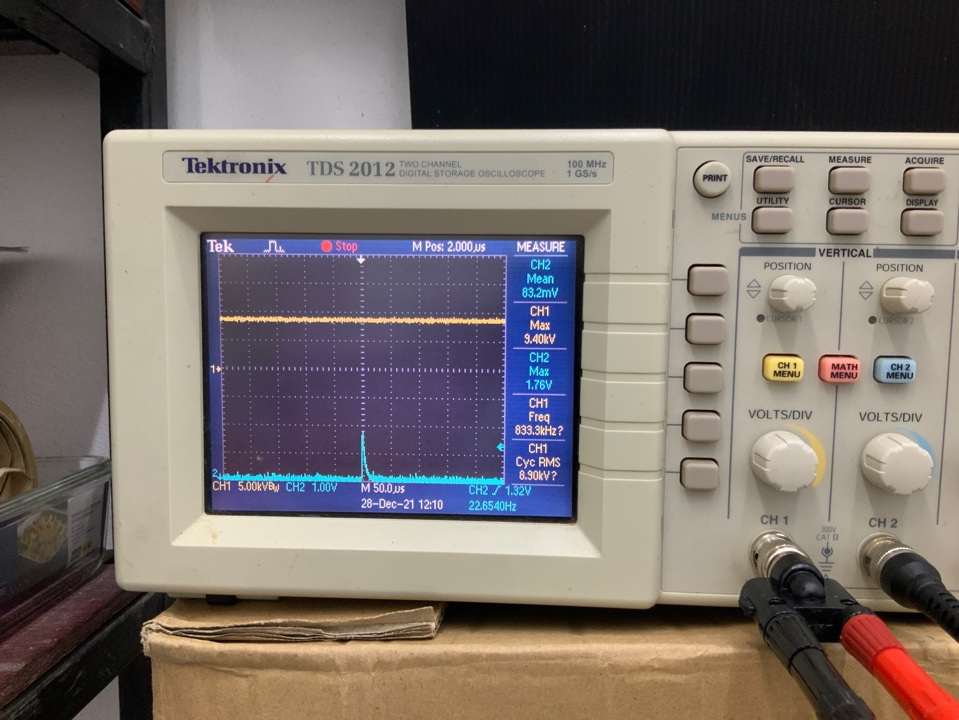  Vd = 9.4 kV | **16** | **0.02** |
| **(b)** | 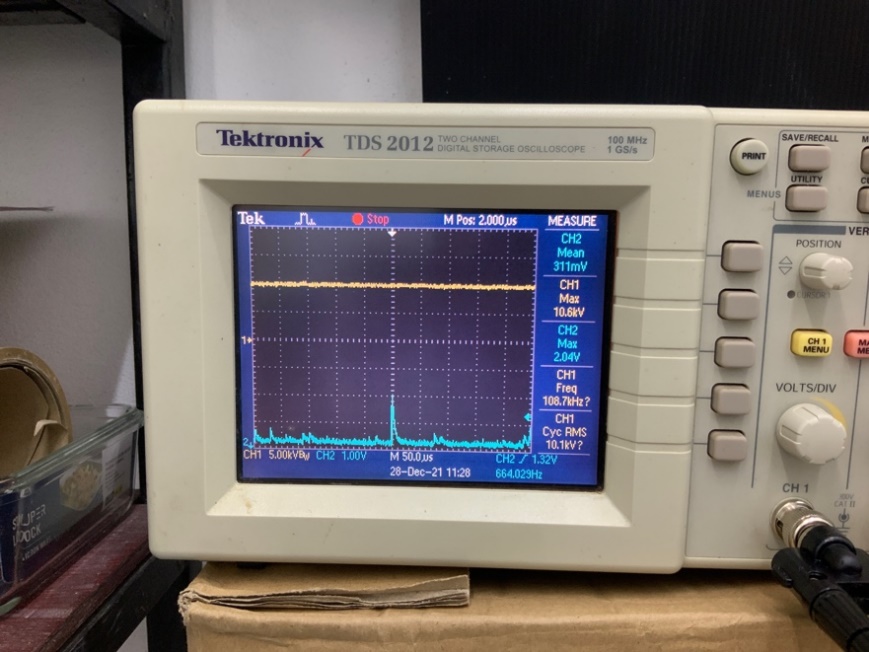  Vd = 10.6 kV | **18** | **0.03** |
| **(c)** | 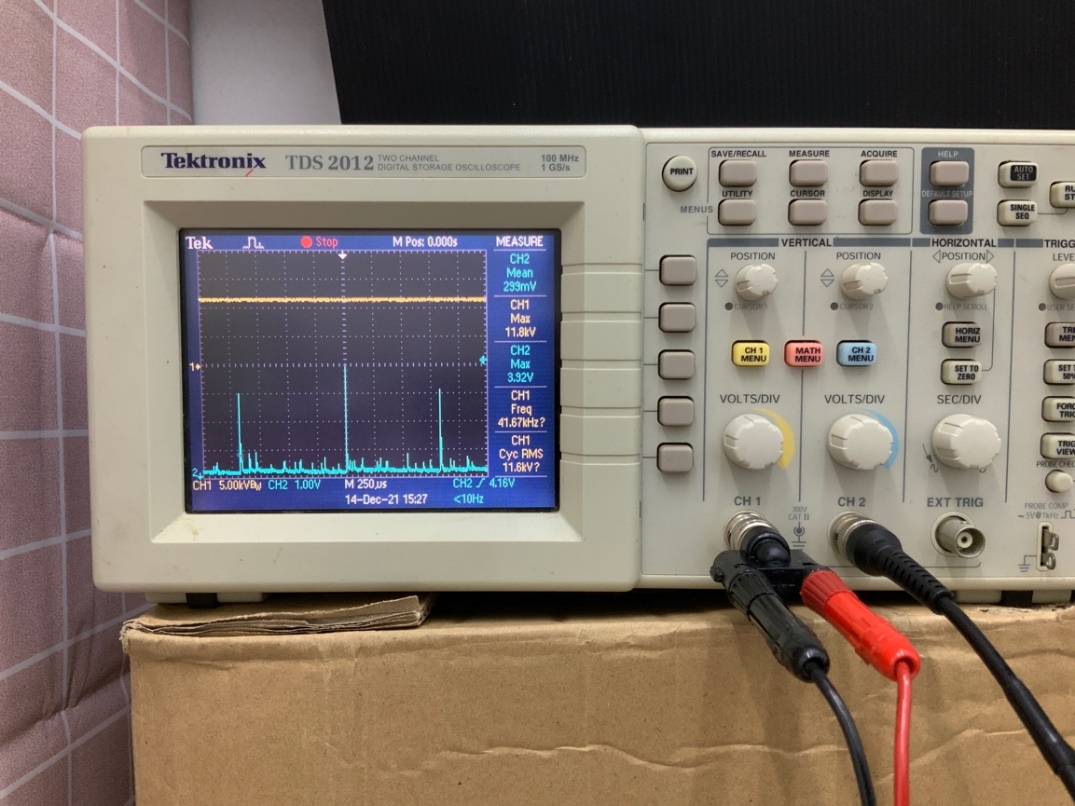  Vd = 11.8 kV | **20** | **0.03** |
| **(d)** | 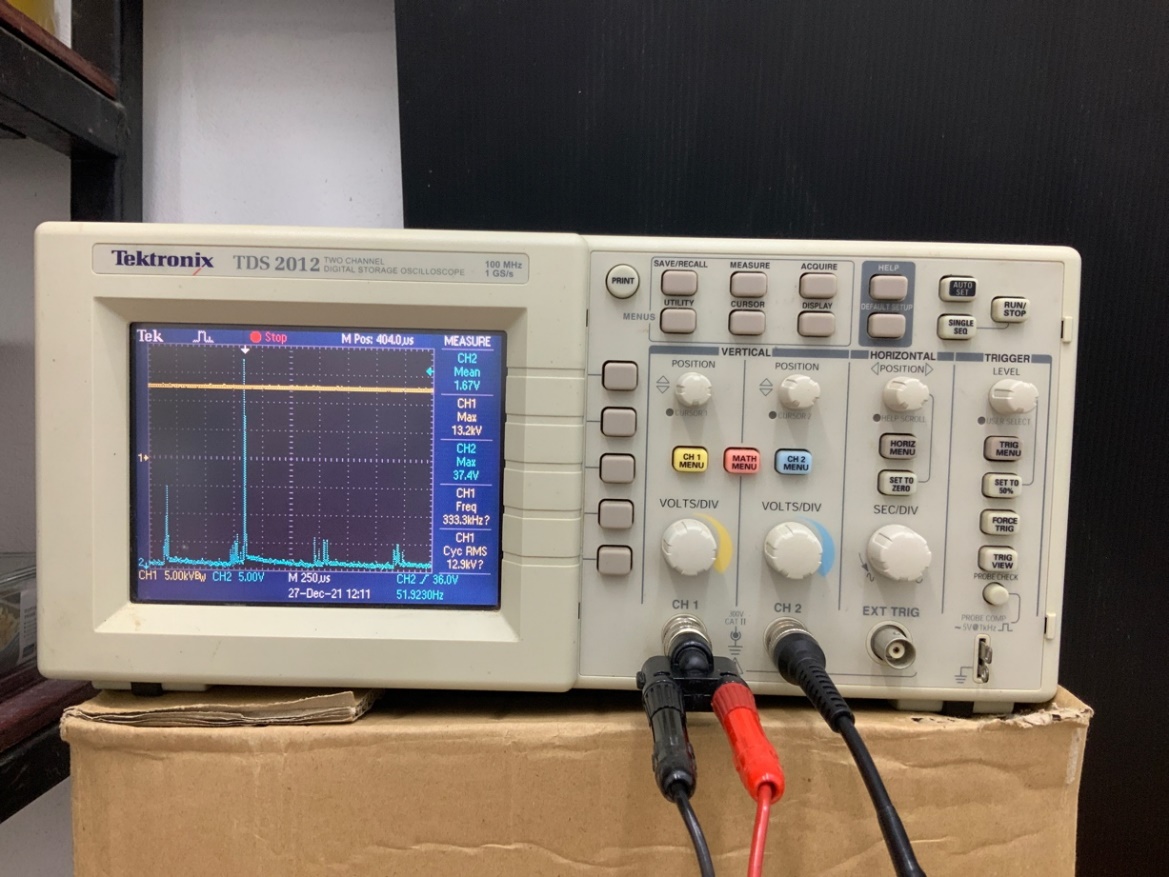  Vd = 13.2 kV | **22** | **0.07** |
| **(e)** | 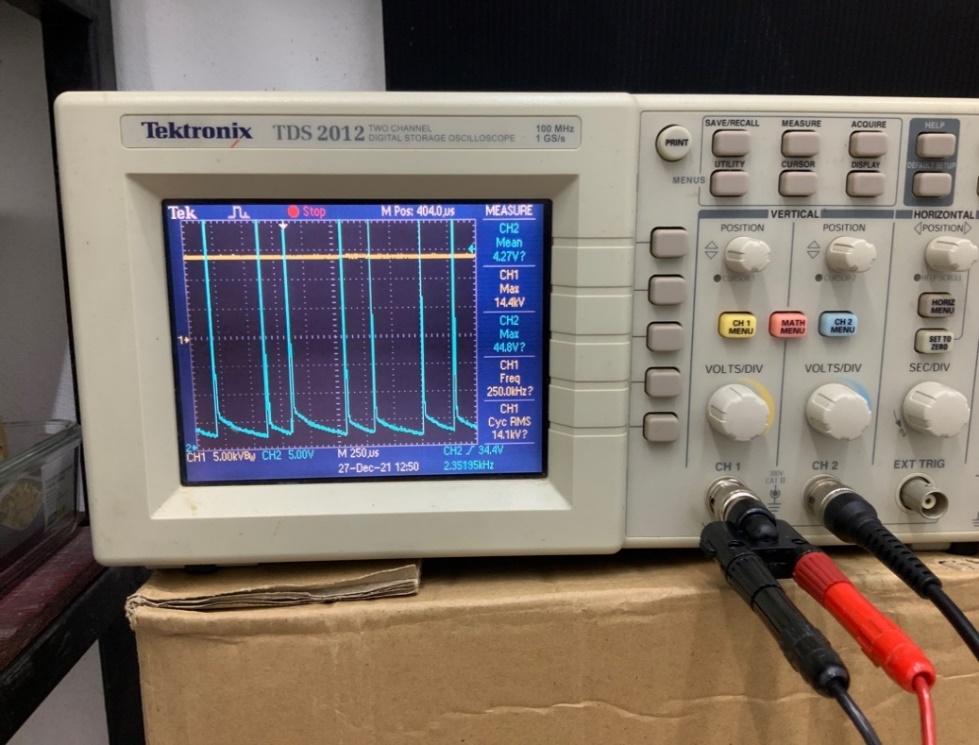  Vd = 14.4 kV | **24** | **0.19** |

**Table S2.** V-I signals of blue printed paper

| **No** | **DSO** | **HVDC**  **(kV)** | **Applied current (mA)** |
| --- | --- | --- | --- |
| **(a)** | **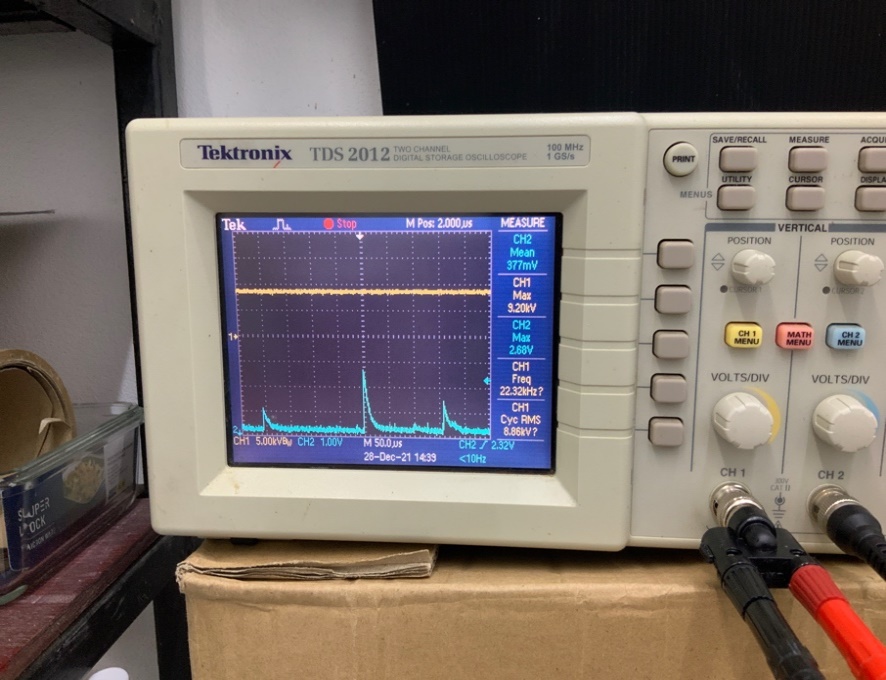**  Vd = 9.2 kV | **16** | **0.02** |
| **(b)** | **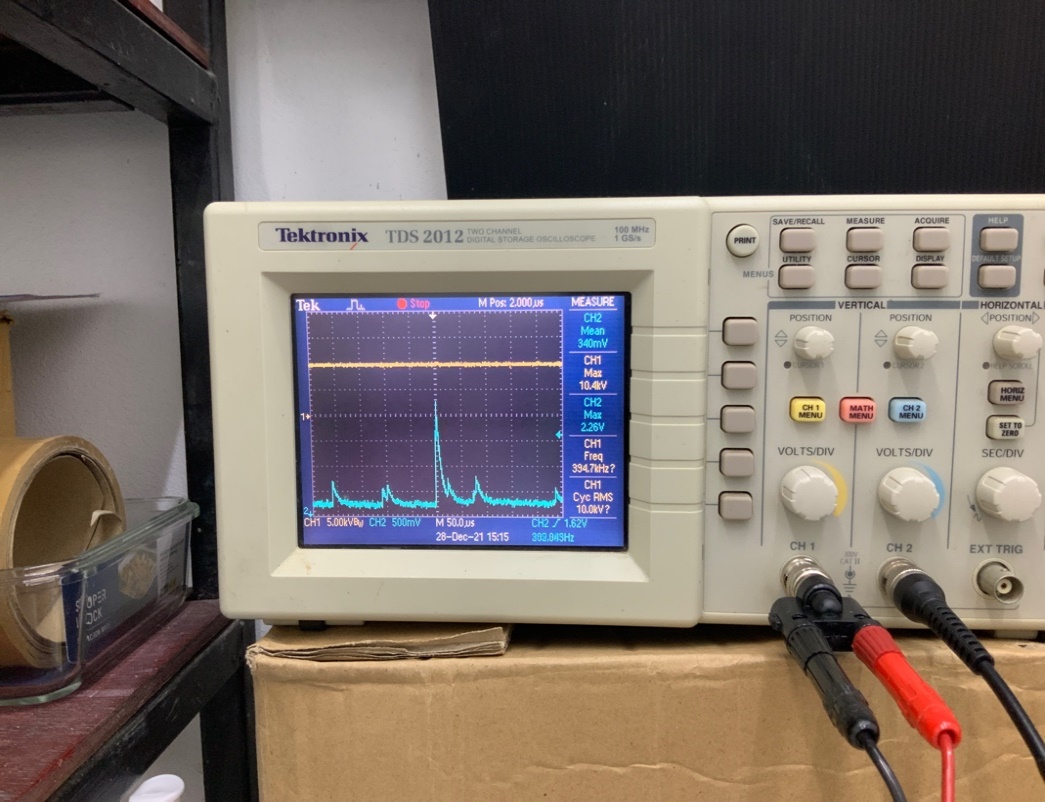**  Vd = 10.4 kV | **18** | **0.02** |
| **(c)** | **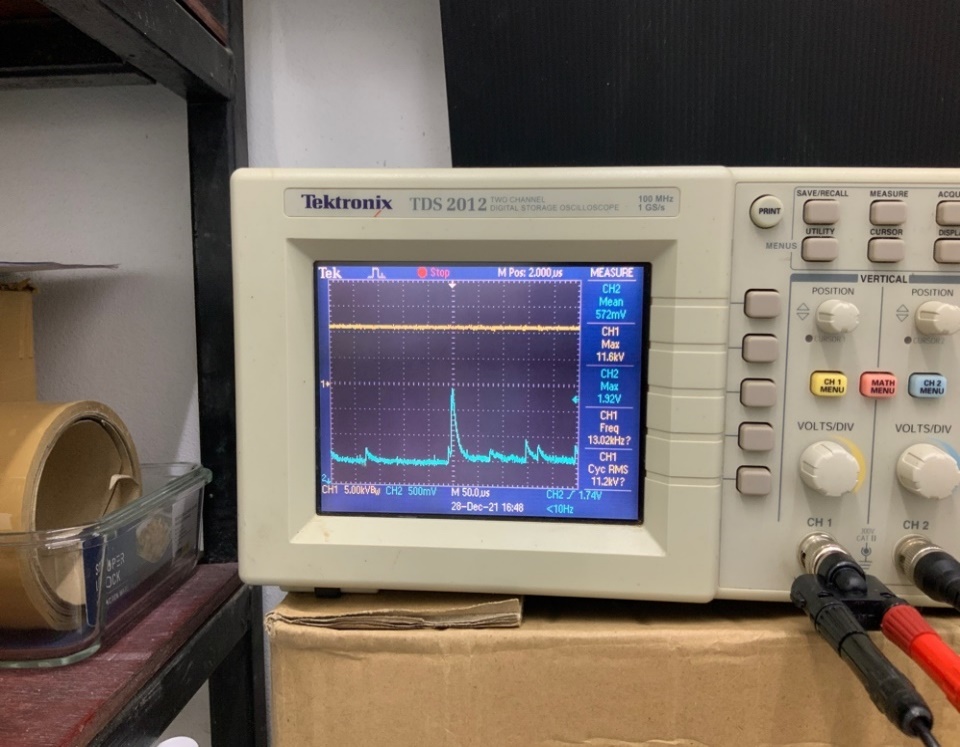**  Vd = 11.6 kV | **20** | **0.03** |
| **(d)** | **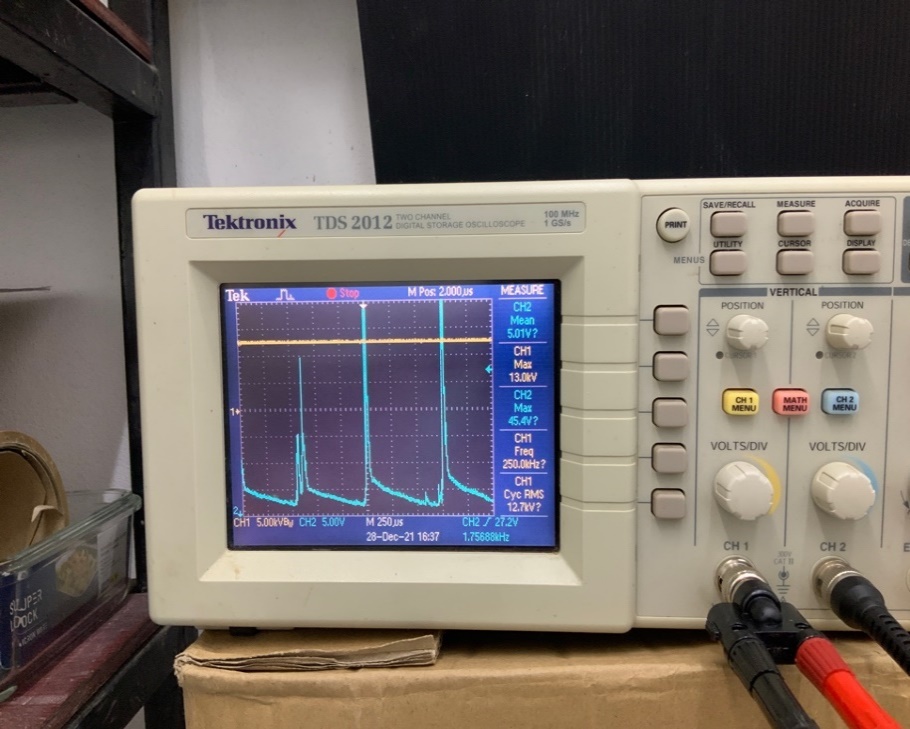**  Vd = 13.0 kV | **22** | **0.1** |
| **(e)** | **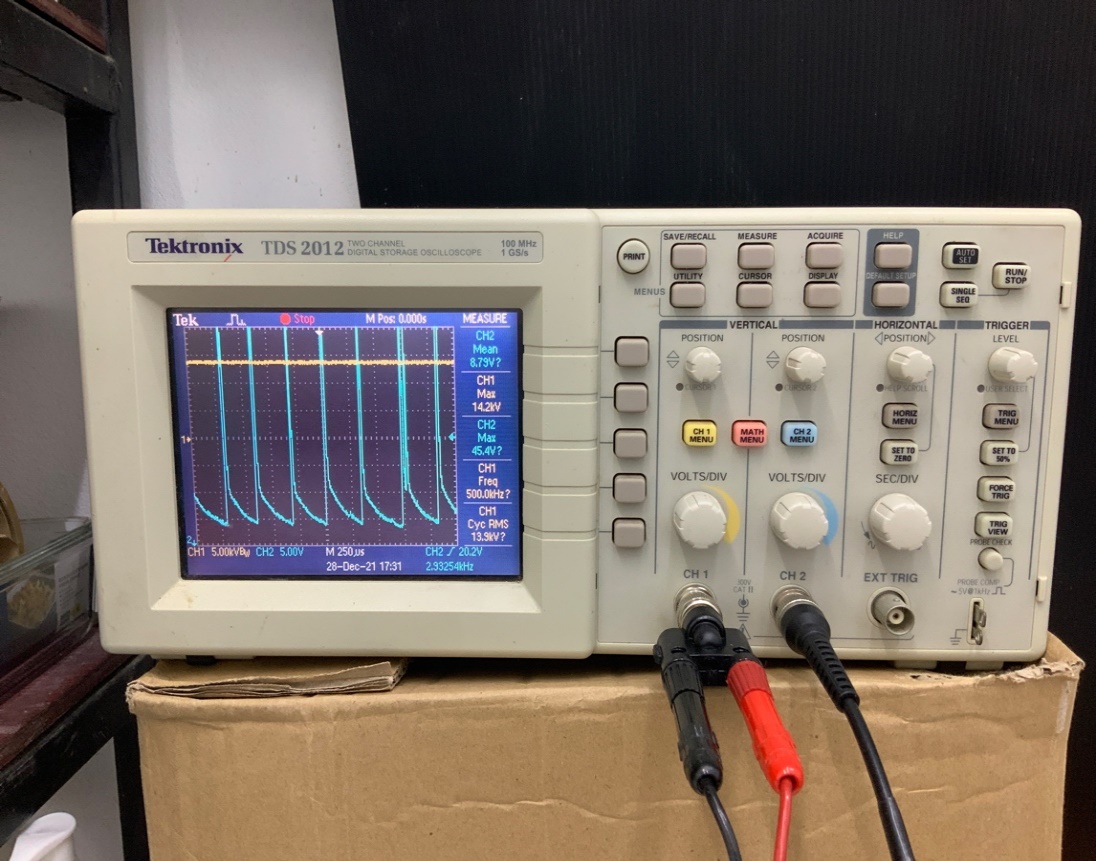**  Vd = 14.2 kV | **24** | **0.28** |

**Table S3**. V-I signals of red printed paper

| **No** | **DSO** | **HVDC**  **(kV)** | **Applied current (mA)** |
| --- | --- | --- | --- |
| **(a)** | **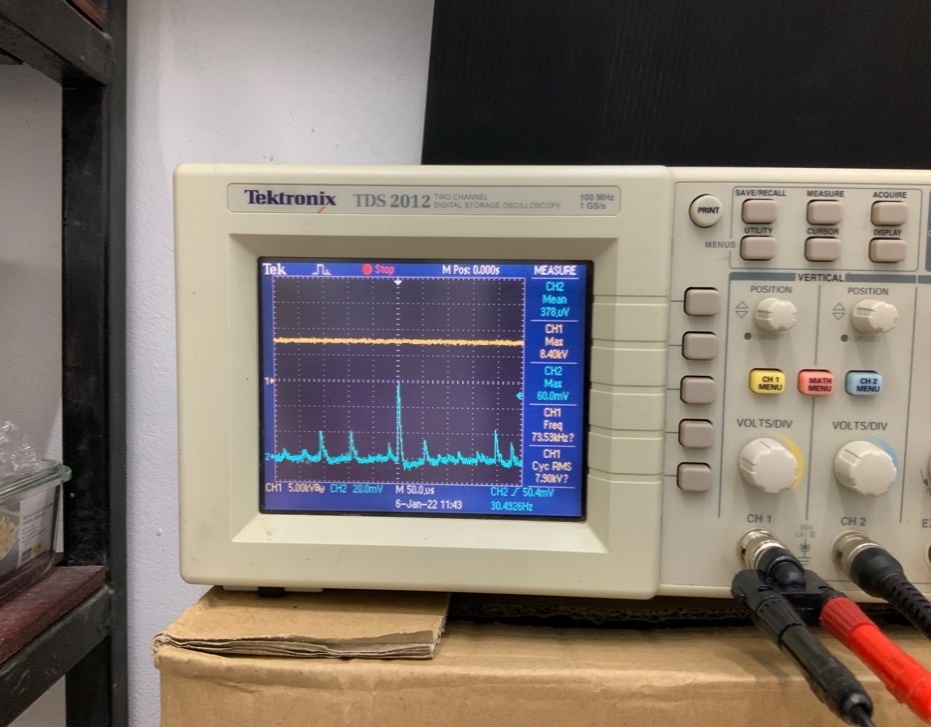**  Vd = 8.4 kV | **16** | **0.03** |
| **(b)** | **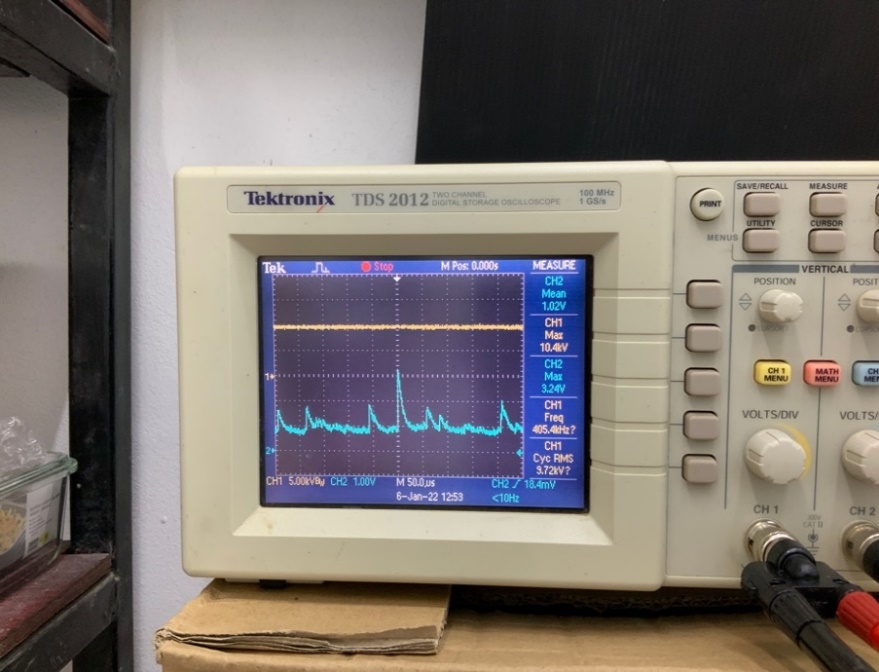**  Vd = 10.4 kV | **18** | **0.03** |
| **(c)** | **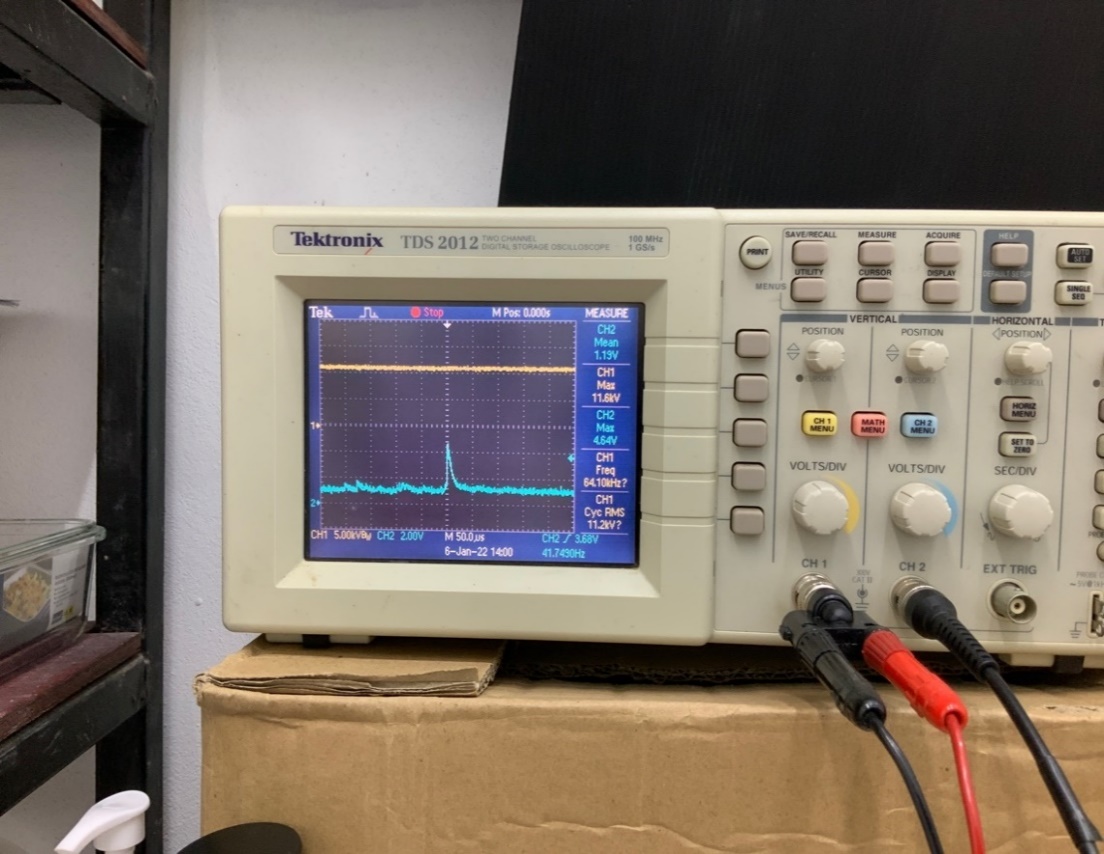**  Vd = 11.6 kV | **20** | **0.04** |
| **(d)** | **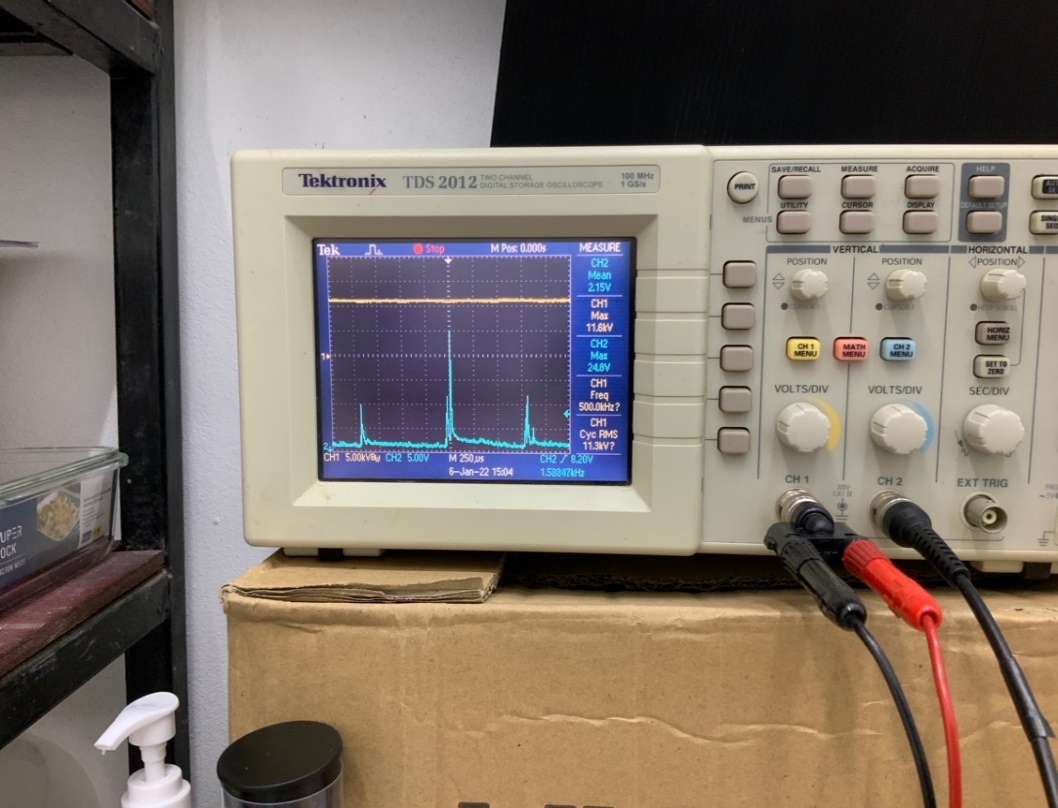**  Vd = 11.6 kV | **22** | **0.07** |
| **(e)** | **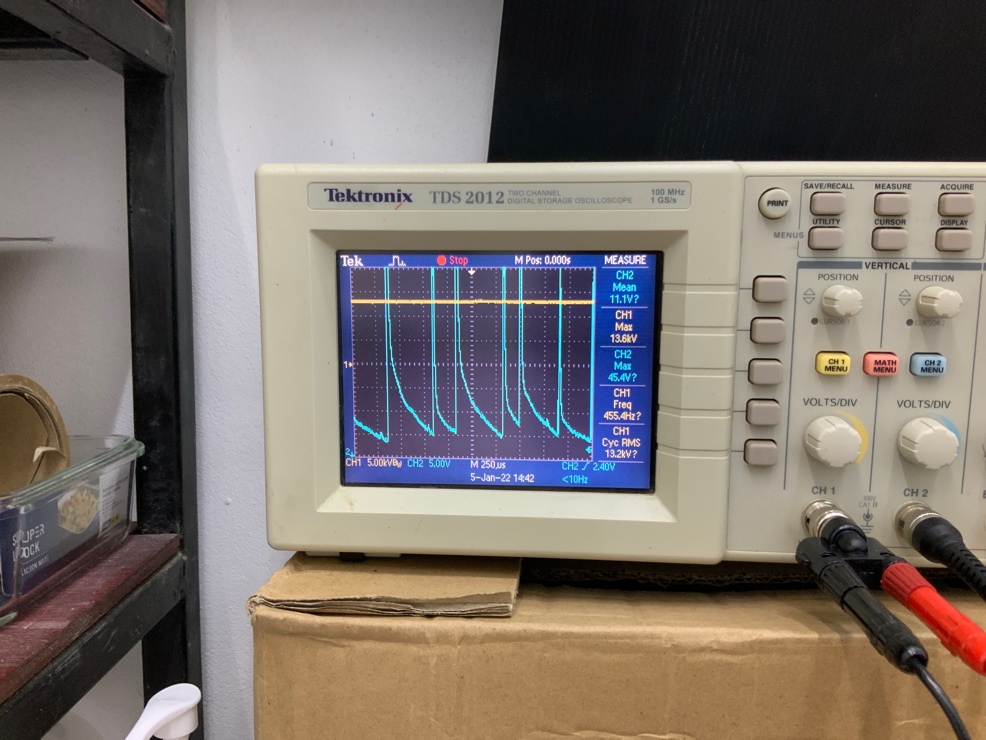**  Vd = 13.6 kV | **24** | **0.25** |

**Table S4. Observed discharged current and discharge time of corona plasma for all colors of printed paper (HVDC of 16-24 kV).**

| **No.** | **Applied voltage**  **(kV)** | **DSO** | **Observed discharge current (A)** | ***Discharge time**  **(µs)** |
| --- | --- | --- | --- | --- |
| **1** | **16** | **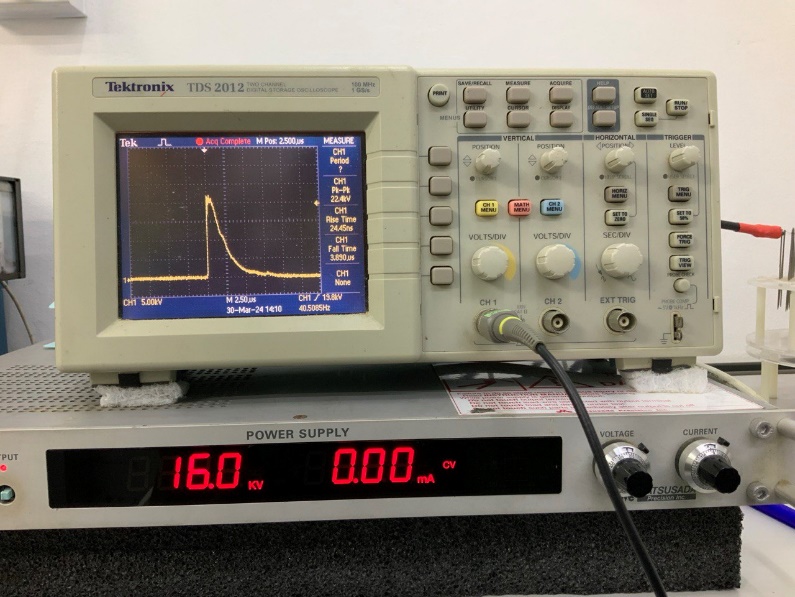** | **I_d_ = V/R**  **I_d_ = 22.4 kV/33kΩ**  **= 0.67** | **6** |
| **2** | **18** | **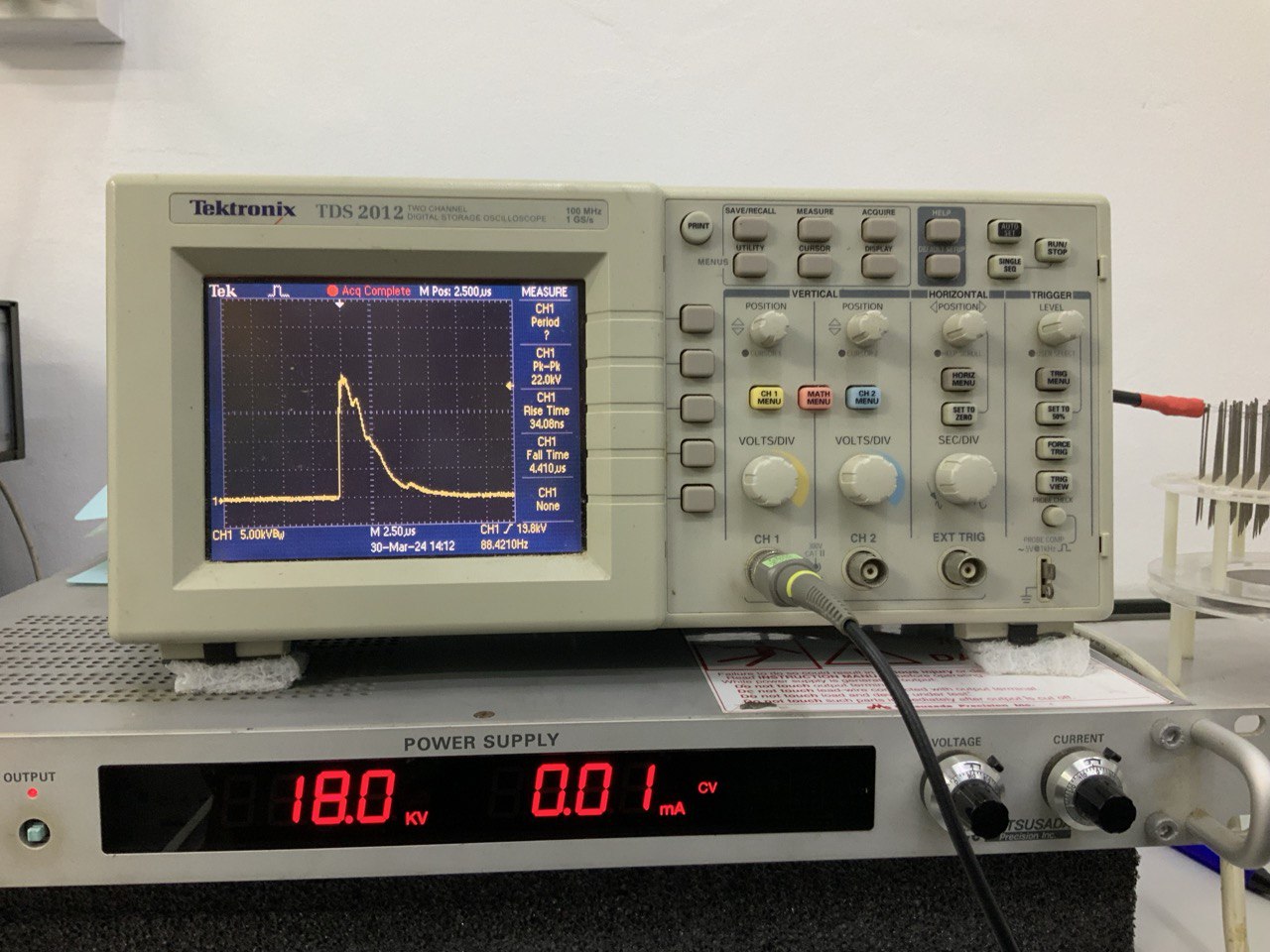** | **0.67** | **7** |
| **3** | **20** | **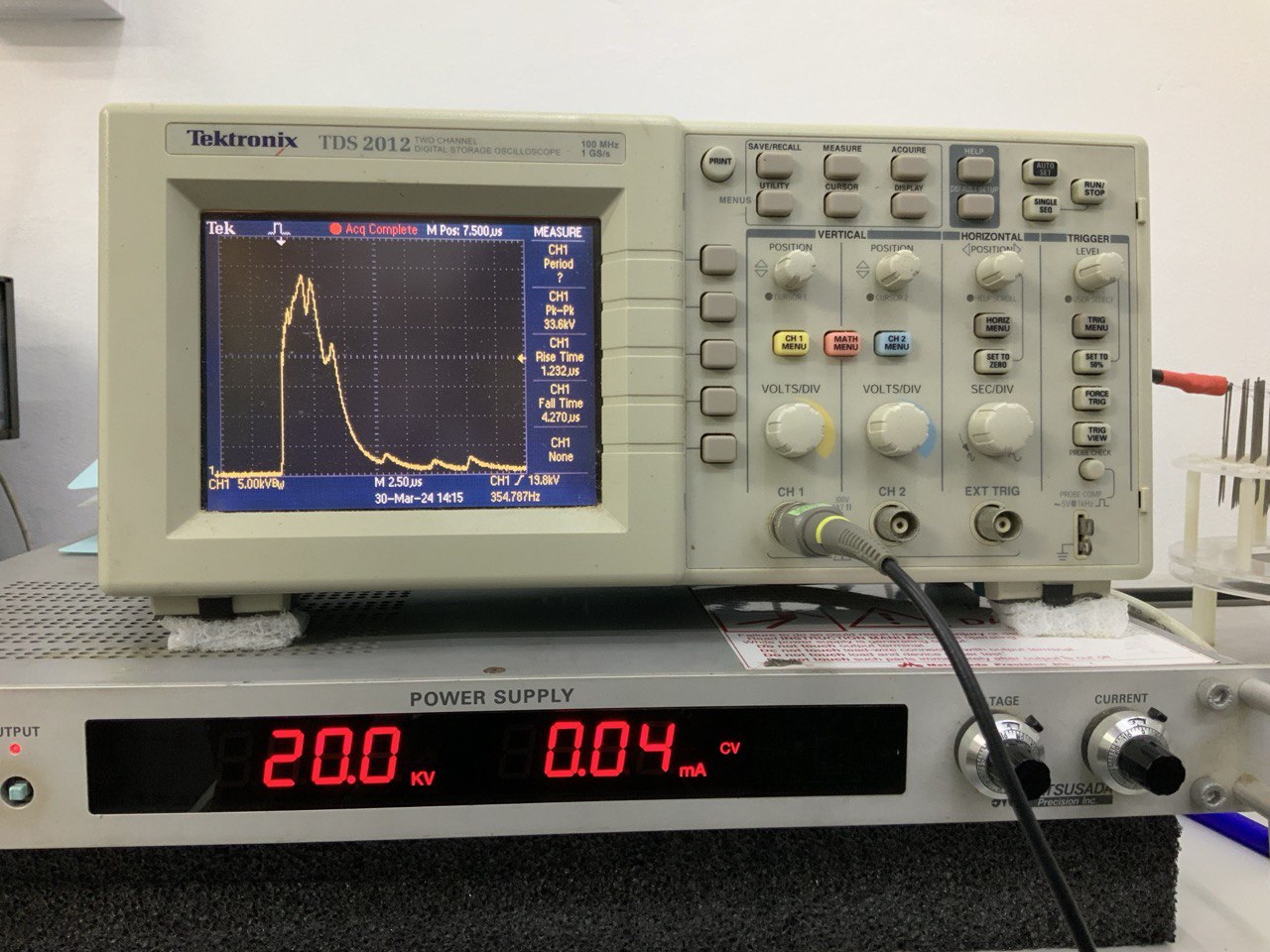** | **1.01** | **15** |
| **4** | **22** | **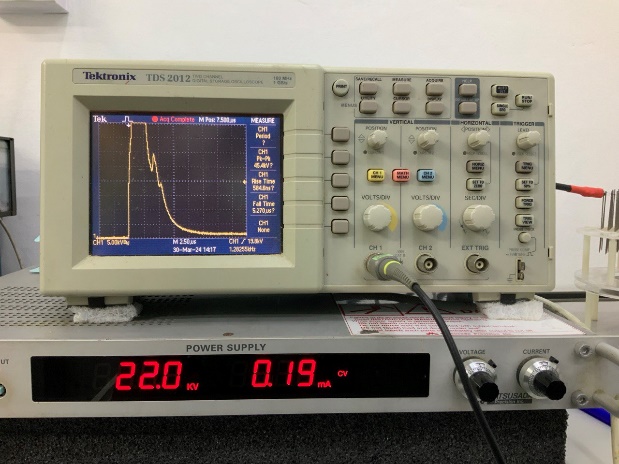** | **1.37** | **Unidentified.**  **Signal peak was clipped due to instrument limitation.** |
| **5** | **24** | **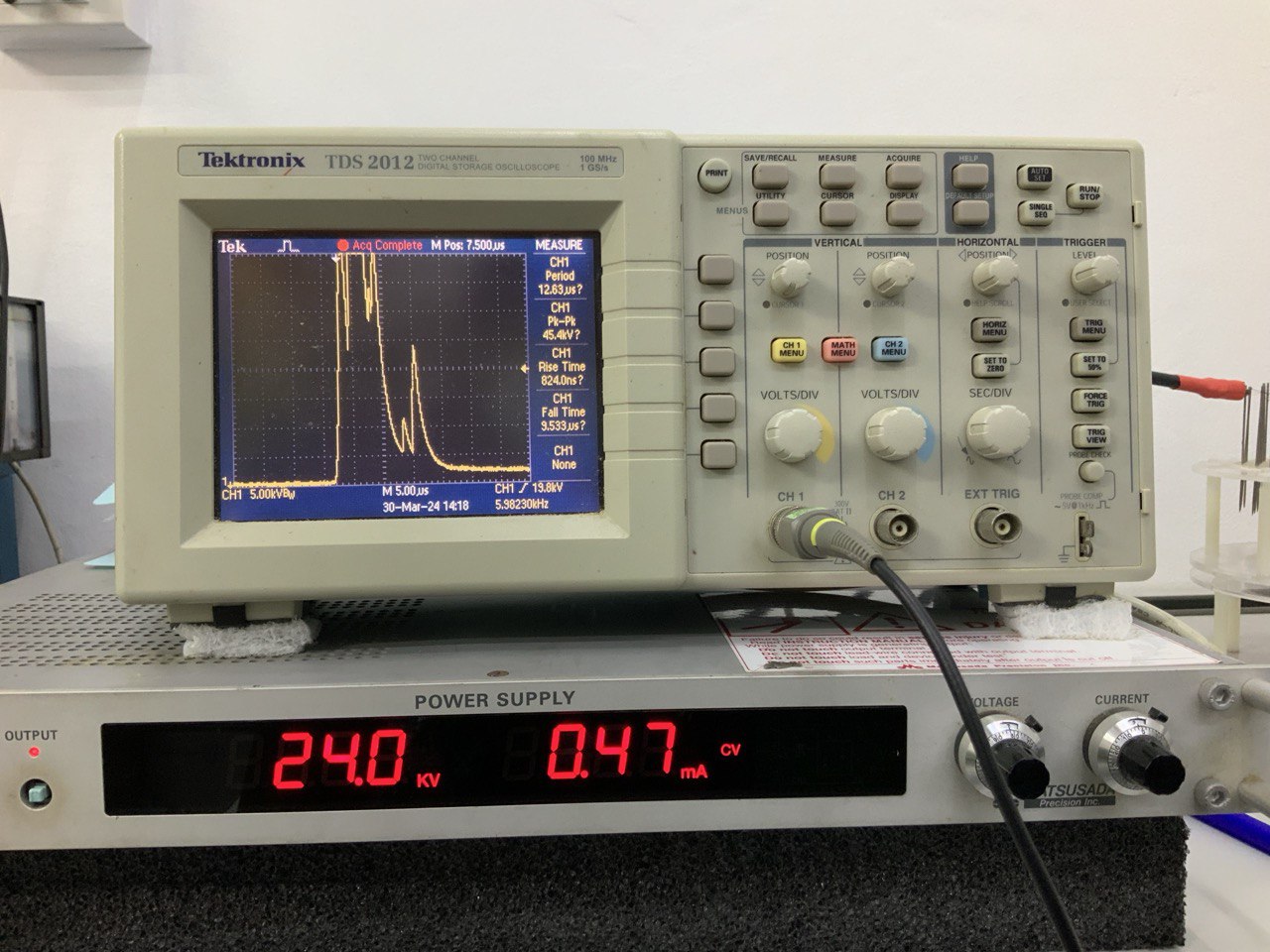** | **1.37** | **Unidentified.**  **Signal peak was clipped due to instrument limitation.** |

| ** | ** |
| --- | --- |
| *(a)* | *(b)* |

**Fig. S1***.* L*a*b color change before **(a)** and after immersion **(b)** of yellow paper

|  |  |
| --- | --- |
| *(a)* | *(b)* |

**Fig. S2.** L*a*b color change before **(a)** and after immersion **(b)** of blue paper

|  |  |
| --- | --- |
| *(a)* | *(b)* |

**Fig. S3***.* L*a*b color change before **(a)** and after immersion **(b)** of red paper
